# Correlates of circulating ovarian cancer early detection markers and their contribution to discrimination of early detection models: results from the EPIC cohort

**DOI:** 10.1186/s13048-017-0315-6

**Published:** 2017-03-20

**Authors:** Renée T. Fortner, Allison F. Vitonis, Helena Schock, Anika Hüsing, Theron Johnson, Raina N. Fichorova, Titilayo Fashemi, Hidemi S. Yamamoto, Anne Tjønneland, Louise Hansen, Kim Overvad, Marie-Christine Boutron-Ruault, Marina Kvaskoff, Gianluca Severi, Heiner Boeing, Antonia Trichopoulou, Vassiliki Benetou, Carlo La Vecchia, Domenico Palli, Sabina Sieri, Rosario Tumino, Giuseppe Matullo, Amalia Mattiello, N. Charlotte Onland-Moret, Petra H. Peeters, Elisabete Weiderpass, Inger Torhild Gram, Mie Jareid, J. Ramón Quirós, Eric J. Duell, Maria-Jose Sánchez, María Dolores Chirlaque, Eva Ardanaz, Nerea Larrañaga, Björn Nodin, Jenny Brändstedt, Annika Idahl, Kay-Tee Khaw, Naomi Allen, Marc Gunter, Mattias Johansson, Laure Dossus, Melissa A. Merritt, Elio Riboli, Daniel W. Cramer, Rudolf Kaaks, Kathryn L. Terry

**Affiliations:** 1 Division of Cancer Epidemiology, German Cancer Research Center (DKFZ), Im Neuenheimer Feld 280, Heidelberg, 69120 Germany; 2 Ob/Gyn Epidemiology Center, Brigham and Women’s Hospital, Boston, MA USA; 3 Obstetrics, Gynecology and Reproductive Biology, Harvard Medical School, Boston, MA USA; 4 Laboratory of Genital Tract Biology, Brigham and Women’s Hospital, Boston, MA USA; 5 Unit of Diet, Genes and Environment, Danish Cancer Society Research Center, Copenhagen, Denmark; 6 Department of Public Health, Section for Epidemiology, Aarhus University, Aarhus, Denmark; 7 INSERM, Centre for Research in Epidemiology and Population Health (CESP), U1018, Nutrition, Hormones and Women’s Health team, Villejuif, F-94805 France; 8 Université Paris Sud, UMRS 1018, Villejuif, F-94805 France; 9 Gustave Roussy, Villejuif, F-94805 France; 10 Human Genetics Foundation (HuGeF), Torino, Italy; 11 Department of Epidemiology, German Institute of Human Nutrition Potsdam-Rehbruecke, Nuthetal, Germany; 12 Hellenic Health Foundation, Athens, Greece; 13 WHO Collaborating Center for Nutrition and Health, Unit of Nutritional Epidemiology and Nutrition in Public Health, Dept. of Hygiene, Epidemiology and Medical Statistics, School of Medicine, National and Kapodistrian University of Athens, Athens, Greece; 14 Department of Clinical Sciences and Community Health, Università degli Studi di Milano, Milan, Italy; 15 Cancer Risk Factors and Life-Style Epidemiology Unit, Cancer Research and Prevention Institute – ISPO, Florence, Italy; 16 Epidemiology and Prevention Unit, Fondazione IRCCS Istituto Nazionale dei Tumori, Milan, Italy; 17 Cancer Registry and Histopathology Unit, “Civic - M.P-Arezzo” Hospital, ASP, Ragusa, Italy; 18 Department of Medical Sciences, University of Torino and Human Genetics Foundation – HuGeF, Torino, Italy; 19 Dipartimeno di Medicina Clinica e Chirurgia, Federico II University, Naples, Italy; 20 Department of Epidemiology, Julius Center for Health Sciences and Primary Care, University Medical Center Utrecht, Utrecht, Netherlands; 21 MRC-PHE Centre for Environment and Health, Department of Epidemiology and Biostatistics, School of Public Health, Imperial College, London, UK; 22 Department of Community Medicine, Faculty of Health Sciences, University of Tromsø, The Arctic University of Norway, Tromsø, Norway; 23 Department of Research, Cancer Registry of Norway, Institute of Population-Based Cancer Research, Oslo, Norway; 24 Department of Medical Epidemiology and Biostatistics, Karolinska Institutet, Stockholm, Sweden; 25 Genetic Epidemiology Group, Folkhälsan Research Center, Helsinki, Finland; 26 Public Health Directorate, Asturias, Spain; 27 Cancer Epidemiology Research Program, Bellvitge Biomedical Research Institute (IDIBELL), Catalan Institute of Oncology (ICO), Barcelona, Spain; 28 Escuela Andaluza de Salud Pública, Instituto de Investigación Biosanitaria ibs.GRANADA, Hospitales Universitarios de Granada/Universidad de Granada, Granada, Spain; 29 CIBER de Epidemiología y Salud Pública (CIBERESP), Madrid, Spain; 30 Department of Epidemiology/Murcia Health Authority, Murcia, Spain; 31 IMIB-Arrixaca, Murcia University, CIBERESP, Murcia, Spain; 32 Navarra Public Health Institute, Pamplona, Spain; 33 IdiSNA, Navarra Institute for Health Research, Pamplona, Spain; 34 Public Health Division and BioDonostia Research Institute and CIBERESP, Basque Regional Health Department, San Sebastian, Spain; 35 Department of Clinical Sciences, Lund University, Lund, Sweden; 36 Division of Surgery, Skåne University Hospital, Lund, Sweden; 37 Department of Clinical Sciences, Obstetrics and Gynecology, Umeå University, Umeå, Sweden; 38 Cancer Epidemiology Unit, University of Cambridge, Cambridge, United Kingdom; 39 Clinical Trial Service Unit and Epidemiological Studies Unit, Nuffield Department of Population Health, University of Oxford, Oxford, UK; 40 International Agency for Research on Cancer, Lyon, France; 41 Department of Epidemiology and Biostatistics, School of Public Health, Imperial College London, London, UK

**Keywords:** Ovarian cancer, Early detection markers, CA125, CA15.3, HE4

## Abstract

**Background:**

Ovarian cancer early detection markers CA125, CA15.3, HE4, and CA72.4 vary between healthy women, limiting their utility for screening.

**Methods:**

We evaluated cross-sectional relationships between lifestyle and reproductive factors and these markers among controls (*n* = 1910) from a nested case-control study in the European Prospective Investigation into Cancer and Nutrition (EPIC). Improvements in discrimination of prediction models adjusting for correlates of the markers were evaluated among postmenopausal women in the nested case-control study (*n* = 590 cases). Generalized linear models were used to calculate geometric means of CA125, CA15.3, and HE4. CA72.4 above vs. below limit of detection was evaluated using logistic regression. Early detection prediction was modeled using conditional logistic regression.

**Results:**

CA125 concentrations were lower, and CA15.3 higher, in post- vs. premenopausal women (*p* ≤ 0.02). Among postmenopausal women, CA125 was higher among women with higher parity and older age at menopause (p_trend_ ≤ 0.02), but lower among women reporting oophorectomy, hysterectomy, ever use of estrogen-only hormone therapy, or current smoking (*p* < 0.01). CA15.3 concentrations were higher among heavier women and in former smokers (*p* ≤ 0.03). HE4 was higher with older age at blood collection and in current smokers, and inversely associated with OC use duration, parity, and older age at menopause (≤ 0.02). No associations were observed with CA72.4. Adjusting for correlates of the markers in prediction models did not improve the discrimination.

**Conclusions:**

This study provides insights into sources of variation in ovarian cancer early detection markers in healthy women and informs about the utility of individualizing marker cutpoints based on epidemiologic factors.

**Electronic supplementary material:**

The online version of this article (doi:10.1186/s13048-017-0315-6) contains supplementary material, which is available to authorized users.

## Background

Mucins CA125 (MUC16) and CA15.3 (MUC1) are membrane-bound, high molecular weight glycoproteins expressed in certain epithelial tissues, as well as some epithelial cancers [1, 2]. CA125 is expressed in >80% of ovarian cancers, while CA15.3 is commonly expressed in breast cancer [2]. Human epididymis protein 4 (HE4) is a member of the whey acidic protein family and is widely expressed in ovarian cancers [3]. CA72.4, is a mucin-like glycoprotein expressed in gastric, breast, and ovarian cancers [4]. Circulating concentrations of CA125, CA15.3, HE4 and CA72.4 have been investigated for ovarian cancer early detection. However, these markers have limited predictive utility for ovarian cancer screening given low sensitivity and specificity for early stage disease, as described in an earlier investigation by our group [5]. Variable circulating concentrations of these markers are found in healthy women, limiting their utility for screening.

Reproductive and lifestyle factors previously shown to impact CA125, CA15.3 and HE4 concentrations in healthy women include age, hysterectomy, oral contraceptive (OC) use, body mass index (BMI; kg/m^2^), and smoking status [6–13]. CA125 concentrations are higher in premenopausal, relative to postmenopausal, women [8, 13–17], and different screening cutpoints have been proposed based on menopausal status (e.g., 98^th^ percentile in healthy women, premenopausal: 52 U/mL; postmenopausal: 35 u/mL [8]).

Given these observations in healthy women, understanding correlates of early detection markers could help improve the utility of these markers in early detection prediction models. We therefore (i) describe associations between lifestyle and reproductive factors and CA125, CA15.3, HE4, and CA72.4; and, (ii) evaluate whether adjusting for these factors in early detection prediction models including early detection marker data improves the discriminatory capacity of these markers in a large, prospective investigation in the European Prospective Investigation into Cancer and Nutrition (EPIC) cohort.

## Methods

The EPIC cohort was established between 1992 and 2000 in 23 centers in 10 European countries: Denmark, France, Germany, Greece, Italy, the Netherlands, Norway, Spain, Sweden, and the United Kingdom. Approximately 500,000 participants were recruited at study baseline. Study participants completed questionnaires describing diet, reproductive history, menstrual factors, exogenous hormone use, as well as disease history, smoking, and alcohol use. A total of 385,747 (74%) participants provided a blood sample at or near baseline. The study was approved by the ethics committee of the International Agency for Research on Cancer (IARC) and the ethical committees at the participating centers.

Details of study design and follow-up have been published previously [18]. Briefly, follow-up is conducted via linkages with cancer and population registries with the exception of centers in Germany, Greece, and Naples, Italy; these centers utilize a combination of active follow-up, next-of-kin, and population registries.

### Study population

Selection of the cases and controls for this nested case-control study has been described in detail previously [5]. Briefly, incident ovarian (*n* = 752), fallopian tube (*n* = 33), and primary peritoneal (*n* = 25) cancers were matched to up to four controls (*n* = 1938) on study recruitment center, age at blood donation (±6 months), time of the day of blood collection (±1 h), fasting status (<3 h, 3–6 h, >6 h), menopausal status at blood collection (premenopausal, perimenopausal, postmenopausal), and current use of exogenous hormones (OC, menopausal hormone therapy (HT)) at the time of blood draw, as well as menstrual cycle phase for premenopausal women (3–5 categories, depending on available data) using incidence density sampling.

The primary cross-sectional analyses included pre- and postmenopausal controls from the nested case-control study (*n* = 1910). Given established differences in circulating CA125 by menopausal status [8], cross-sectional analyses were restricted to women pre- or postmenopausal at time of blood collection. Women were considered premenopausal if they met one of the following criteria at blood collection: menstruated at least once in the prior year while not on hormones; were on hormones but were less than 50 years old; had a hysterectomy before last period and were less than 50 years old; or, age at last menstruation was missing and age was less than 50. Postmenopausal status was assigned to women who met one of the following criteria at blood collection: were not on hormones and had not menstruated in the past year, on hormones and age was 50 or greater, had a hysterectomy and age was 50 or greater, age at last menstruation was missing and age was 50 or greater. Controls that were perimenopausal or had unknown menopausal status (*n* = 28) were excluded. In a secondary analysis, we evaluated cross-sectional associations among cases (*n* = 791). Cases who were perimenopausal or had unknown menopausal status (*n* = 19) were excluded from these analyses.

### Exposure data

Data on lifestyle and reproductive exposures, as well as anthropometric measures, were collected at baseline and included: age at menarche, age at blood draw, OC use and duration, HT use and duration, type of postmenopausal HT, parity, estimated number of ovulatory cycles (defined as the time between age at menopause and age at menarche not taking OCs or pregnant), phase of menstrual cycle at blood collection (premenopausal women), tubal ligation, hysterectomy, oophorectomy, BMI, smoking, and family history of breast cancer. Those missing exposure of interest were excluded from analyses for that exposure. Among controls, the following variables had missing observations: age at menarche (*n* = 78), OC use (*n* = 57), duration of OC use (*n* = 66), parity (*n* = 136), number of children (*n*=43), tubal ligation (*n* = 1661), IUD use at recruitment (*n* = 522), hysterectomy (*n* = 347), ovulatory cycles (*n* = 385), age at menopause (*n* = 244), HT duration (*n* = 166), HT at blood (*n* = 359), BMI (*n* = 94), smoking (*n* = 28), pack-years among smokers (*n* = 10), and family history of breast cancer (*n* = 1250).

### Laboratory methods

All assays were performed in the Brigham and Women’s Hospital Obstetrics and Gynecology Laboratory of Genital Tract Biology using a volume-efficient highly sensitive multiplex platform (Meso Scale Discovery (MSD), Gaithersburg, MD, USA) based on electrochemiluminescence (ECL) detection. Single ECL assays for antigen detection of human CA125 (catalog number K151WC) and Human Prototype CA15.3 (Catalog number N45ZA-1) and all reagents related to these two assays were provided by MSD. The linearity range for CA125 was 0.6–10,000 U/ml, and for CA15.3 was 0.19–12,500 mU/ml. HE4 and CA72.4 were analyzed using a custom-designed duplex assay. The following reagents were provided by Fujirebio Diagnostics, Inc. (Malvern, PA): HE4 protein (IgHE4 antigen), which we used to generate a calibration curve with a linear range of 0.0137–3600 pM; anti-HE4 capture IgG1 (2H5 mouse hybridoma); anti-HE4 detection IgG1 (mouse hybridoma 3D8); TAG72 Defined Antigen, which we used to generate a calibrator curve with a linear range of 0.146–2400 U/ml; anti-CA72.4 capture IgG1 (mouse hybridoma CC49, Fujirebio catalog number 110–005); anti-CA72.4 detection IgG1 (mouse hybridoma B72.3). The samples were split into batches such that matched case-control sets and samples from the same study center were kept together in the same batches. The samples were tested undiluted in the CA125 singleplex and the HE4/CA72.4 duplex, and they were tested at a 50-fold dilution in the CA15.3 assay. Blinded quality control (QC) samples were included on each assay plate. In blinded QCs with values within the linearity range of each assay we observed the following interplate CVs and min-max (mean) intraplate CVs: 19% and 3–20 (9)% for CA125, 22% and 3–5% (4%) for CA15.3, 9% and 4–10% (6)% for HE4, 16% and 1–16% (6%) for CA72.4). CA72.4 concentrations were below the lower limit of detection in the blinded QC samples, therefore CVs are based on the remaining 13 aliquots (concentration range: 1.15 to 1.87 U/mL).

### Statistical analyses

Biomarker concentrations were log-transformed to obtain a more normal data distribution. We assessed each biomarker for outliers using the generalized extreme studentized deviate many-outlier procedure [19]. Eight outliers were identified for HE4; the influence of these values was assessed in sensitivity analyses. No outliers were identified for CA125 or CA15.3. We used generalized linear models to estimate the mean CA125, CA15.3, and HE4 values across categories of each characteristic and exponentiated results to obtain geometric mean values in the original scale. Since the majority of the CA72.4 values (82%) were below the lower detection limit (1.119 U/mL), we used a logistic regression analysis with a dichotomous CA72.4 variable (≥1.119 vs. < 1.119 U/mL) as the outcome and results are presented only in a supplemental table. Wald tests of continuous variables were used to assess trend. All analyses were adjusted for matching factors from the parent nested case–control study: study center (grouped by country), age at blood draw, fasting status, date of blood draw, menstrual cycle phase for women premenopausal at blood collection, OC/HT use at blood collection, and length of follow up. We adjusted for oophorectomy, number of ovulatory cycles, and smoking status in sensitivity analyses among premenopausal women, and these factors plus age at menopause, hysterectomy, and type of HT among postmenopausal women. Missing indicators were used to account for missing data for covariates. CA125 and CA15.3 have been reported to vary across the menstrual cycle [20, 21]. Therefore, we evaluated these markers both adjusting for menstrual cycle phase and standardized using phase-specific residuals. Results were similar with both approaches; we present the models adjusted for menstrual cycle phase.

To assess whether the adjustment for correlates of these early detection markers improved discrimination between controls and individuals who subsequently became cases, we evaluated the area under the receiver operating characteristic curve (AUC) and compared AUCs from models including the marker alone to those including the marker standardized for its correlates. These analyses were limited to cases who were postmenopausal at time of blood collection (*n* = 590; and their matched controls), given significant predictors of the markers were only identified among women postmenopausal at blood collection. AUCs were calculated using conditional logistic regression models to account for the matched study design. We calculated absolute risk estimates for ovarian cancer using a model derived in the EPIC cohort [22] and calibrated the conditional logistic regression model towards the absolute risk estimates as an offset variable. We used regression residuals to standardize the marker concentrations based on significant correlates of the marker. Briefly, we calculated the deviation (residual) from the mean predicted concentration given each study participant’s profile of correlates. Correlates included for each marker were: CA125: parity, hysterectomy, unilateral oophorectomy, age at menopause, estrogen-alone HT use, ovulatory cycles, current smoking; CA15.3: BMI, former smoking; HE4: age at blood draw, OC use, parity, age at menopause, current smoking; CA72.4: no correlates identified.

Analyses were conducted using SAS version 9.4 (Cary, NC) and R 3.3.0. All statistical tests were two-tailed and significant at *p* < 0.05.

## Results

Study participants in the primary cross-sectional analyses restricted to controls were mean age 56 years at blood collection, and 74% were postmenopausal (*n* = 1421; premenopausal, *n* = 489). The majority of participants were parous (89%), half were ever users of OCs at the time of blood collection, and 33% of postmenopausal women reported using HT use at the time of blood collection. Average BMI was 25.8 kg/m^2^, and 19% reported smoking at the time of blood draw (Table 1). Characteristics of the full nested case-control study population have been presented previously [5]. Briefly, cases were median age 63 years at diagnosis (range: 31–86 years), with median 6 years between blood collection and diagnosis (range: 0–16 years). The majority of cases were diagnosed with tumors of serous histology (*n* = 443; 55%).

**Table 1:** Population characteristics for controls included in cross sectional analysis of CA125, CA15.3, HE4, and CA72.4: EPIC ovarian cancer nested case-control study

Characteristic	Total sample *n* = 1910
Age at blood draw, mean (sd)	56.3 (8.3)
Age at menarche, mean (sd)	13.3 (1.6)
Ever OC use, n (%)	930 (50%)
OC duration among users, years, mean (sd)	8.5 (8.1)
Parous, n (%)	1573 (89%)
Number of children among parous, mean (sd)	2.4 (1.1)
Unilateral oophorectomy, n(%)	70 (4%)
Postmenopausal, n (%)	1421 (74%)
HT use among postmenopausal women	463 (33%)
BMI, kg/m^2^, mean (sd)	25.8 (4.5)
Current smoker, n (%)	360 (19%)
Packyears of smoking among current smokers, mean (sd)	21.4 (13.5)
CA125 (U/mL), geometric mean (95% CI)	20.1 (19.6, 20.7)
CA15.3 (mU/mL), geometric mean (95% CI)	600.4 (585.7, 615.5)
HE4 (pM), geometric mean (95% CI)^a^	18.9 (18.2, 19.5)
CA72.4 (U/mL), geometric mean (95% CI)^a^	0.69 (0.66, 0.72)

Some participants had missing data: age at menarche (*n* = 78), oral contraceptive use (*n* = 57), duration of OC use (*n* = 66), parity (*n* = 136), number of children (*n* = 43), BMI (*n* = 94), smoking (*n* = 28), packyears among current smokers (*n* = 10), CA125 (*n* = 10), CA15.3 (*n* = 17), HE4 (*n* = 1197), CA72.4 (*n* = 1197)

^a^ Restricted to 713 controls with HE4/CA72.4 measurements. Note, participants with CA72.4 less than the limit of detection were assigned value of 0.56 (half of the limit of detection); geometric mean (95% CI) retricted to women with values above the limit of detection: 2.4 (2.0-2.9)

**Fig. 1:**
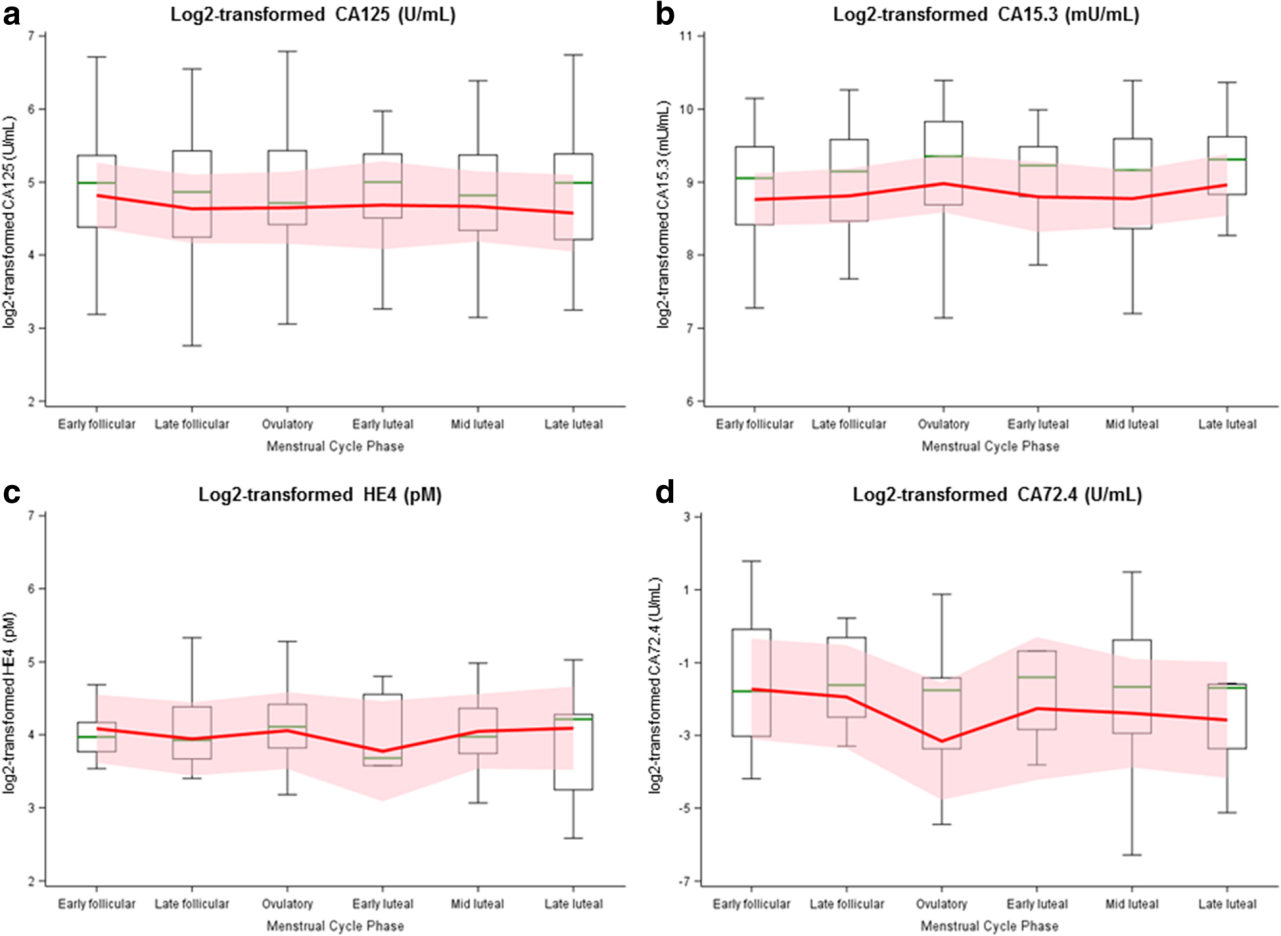
Variation of markers across the menstrual cycle. Box plots and geometric means (*red line*) and 95% confidence intervals (*red cloud*) for CA125 (panel **a**), CA15.3 (panel **b**), HE4 (panel **c**), and CA72.4 (panel **d**; among women with concentrations > LOD)

CA125 concentrations differed significantly by menopausal status at blood collection, with lower concentrations observed among postmenopausal women (premenopausal: 26.1 U/mL; postmenopausal: 18.4 U/mL; *p* < 0.01; Table 2). Concentrations of CA15.3 were significantly higher among postmenopausal (617.5 U/mL) compared to premenopausal (552.9 U/mL, *p* = 0.02) women. HE4 concentrations did not differ by menopausal status at blood collection (*p* = 0.92). Among premenopausal women, biomarker concentrations did not differ significantly by menstrual cycle phase (Fig. 1).

**Table 2:** Association between epidemiologic characteristics and CA125, CA15.3, and HE4 by menopausal status at blood collection in controls: EPIC ^a^

	Premenopausal					Postmenopausal				
		CA125	CA15.3		HE4		CA125	CA15.3		HE4
	*N* ^a^ (%)	Mean (95% CI)^c^	Mean (95% CI)^c^	*N* ^b^ (%)	Mean (95% CI)^c^	*N* ^a^ (%)	Mean (95% CI)^c^	Mean (95% CI)^c^	*N* ^b^ (%)	Mean (95% CI)^c^
Menopausal status^d^										
	485 (26)	26.1 (24.0, 28.3)	552.9 (512.8, 596.1)	175 (25)	19.0 (16.9, 21.3)	1417 (24)	18.4 (17.7, 19.1)	617.6 (596.8, 639.2)	538 (75)	18.8 (17.9, 19.8)
Age at blood draw										
< 41	75 (15)	27.0 (23.1, 31.5)	629.0 (550.6, 718.5)	33 (19)	17.7 (14.4, 21.8)	0 (0)	--	--	0 (0)	--
41–50	269 (55)	29.0 (26.9, 31.3)	560.5 (524.9, 598.6)	103 (59)	17.1 (15.5, 18.7)	47 (3)	18.5 (15.7, 21.9)	532.9 (455.3, 623.8)	9 (2)	17.9 (13.0, 24.6)
51–60	141 (29)	25.4 (22.6, 28.7)	512.3 (462.4, 567.5)	39 (22)	14.5 (12.2, 17.3)	697 (49)	17.8 (17.0, 18.6)	625.4 (600.0, 651.8)	250 (46)	18.4 (17.3, 19.6)
61–70	0 (0)	--	--	0 (0)	--	586 (41)	17.9 (17.1, 18.8)	616.3 (589.3, 644.5)	227 (42)	20.1 (18.8, 21.5)
> 70	0 (0)	--	--	0 (0)	--	87 (6)	21.4 (18.7, 24.4)	596.2 (525.7, 676.0)	52 (10)	25.1 (21.6, 29.2)
p_trend_		0.50	*0.03*		0.18		0.17	0.90		*0.0005*
Oral contraceptive use										
Never	145 (31)	27.8 (25.0, 31.0)	510.1 (465.8, 558.6)	48 (28)	14.7 (12.7, 16.9)	776 (56)	18.2 (17.4, 18.9)	627.7 (602.9, 653.4)	301 (58)	20.0 (18.9, 21.2)
Ever	320 (69)	27.4 (25.6, 29.4)	571.1 (538.7, 605.5)	122 (72)	17.3 (16.0, 18.8)	604 (44)	18.1 (17.2, 19.0)	596.7 (569.7, 625.0)	222 (42)	19.0 (17.8, 20.3)
p_diff_		0.83	*0.05*		0.07		0.88	0.13		0.29
< = 2 years	101 (22)	27.5 (24.3, 31.2)	587.5 (528.8, 652.7)	44 (27)	16.4 (14.2, 18.9)	153 (12)	18.6 (16.9, 20.4)	604.9 (554.4, 660.1)	51 (10)	20.4 (17.9, 23.3)
> 2 to 5 years	68 (15)	29.7 (25.6, 34.5)	532.8 (470.2, 603.8)	22 (13)	19.0 (15.7, 23.1)	111 (8)	17.8 (15.9, 19.8)	578.1 (522.2, 640.0)	44 (9)	18.4 (16.0, 21.1)
> 5 to 10 years	77 (17)	26.1 (22.7, 30.1)	572.9 (508.8, 645.1)	27 (16)	19.1 (15.9, 22.8)	127 (10)	17.2 (15.5, 19.0)	597.2 (542.4, 657.5)	46 (9)	21.5 (18.7, 24.7)
> 10 years	62 (14)	26.7 (22.4, 31.7)	611.6 (528.4, 707.9)	24 (15)	17.2 (13.6, 21.7)	161 (12)	18.7 (17.0, 20.5)	613.3 (562.0, 669.3)	62 (12)	16.3 (14.4, 18.5)
p_trend_		0.55	0.13		0.42		0.71	0.77		*0.006*
p_trend_^ e^		0.67	0.19		0.83		0.68	0.48		*0.02*
Parity										
Nulliparous	48 (11)	30.1 (25.2, 36.0)	561.6 (483.2, 652.6)	18 (11)	14.3 (11.5, 17.7)	152 (12)	16.4 (15.0, 18.0)	605.9 (556.3, 659.9)	62 (12)	22.3 (19.8, 25.0)
Parous	406 (89)	27.4 (25.8, 29.1)	550.1 (523.4, 578.2)	148 (89)	16.8 (15.7, 18.1)	1163 (88)	18.2 (17.6, 18.8)	611.1 (592.6, 630.3)	437 (88)	19.2 (18.4, 20.0)
p_diff_		0.33	0.80		0.17		*0.04*	0.85		*0.02*
1 child	74 (17)	28.3 (24.5, 32.7)	516.8 (458.6, 582.3)	23 (15)	16.4 (13.5, 19.9)	200 (15)	17.2 (15.9, 18.6)	602.8 (558.8, 650.2)	84 (17)	19.7 (17.9, 21.8)
2 children	215 (50)	27.6 (25.4, 30.0)	559.8 (522.1, 600.1)	69 (45)	16.1 (14.4, 18.0)	513 (40)	18.2 (17.3, 19.2)	598.2 (570.6, 627.0)	175 (36)	19.4 (18.1, 20.8)
3 children	75 (17)	26.7 (23.1, 30.8)	556.8 (494.9, 626.4)	38 (25)	17.8 (15.3, 20.7)	270 (21)	18.2 (17.0, 19.5)	623.0 (584.2, 664.4)	106 (22)	19.0 (17.4, 20.8)
4+ children	22 (5)	28.9 (22.1, 37.9)	511.4 (409.6, 638.6)	6 (4)	17.5 (12.0, 25.4)	157 (12)	19.0 (17.3, 20.9)	637.2 (584.3, 695.0)	58 (12)	18.2 (16.1, 20.6)
p_trend_		0.49	0.90		0.19		*0.02*	0.32		*0.02*
p_trend_^ e^		0.81	0.76		0.49		0.16	0.20		0.34
Hysterectomy										
No	411 (97)	27.5 (25.9, 29.2)	550.0 (524.3, 577.0)	154 (98)	16.6 (15.5, 17.7)	972 (86)	18.1 (17.4, 18.7)	591.3 (571.6, 611.8)	364 (86)	19.2 (18.3, 20.2)
Yes	12 (3)	20.0 (13.9, 28.9)	621.8 (460.7, 839.2)	3 (2)	11.0 (6.4, 18.9)	164 (14)	15.5 (14.1, 17.1)	590.3 (540.5, 644.7)	60 (14)	19.3 (17.0, 21.8)
p_diff_		0.10	0.43		0.14		*0.004*	0.97		0.99
Uniliateral oophorectomy										
No	477 (98)	27.7 (26.2, 29.2)	556.8 (531.5, 583.3)	173 (99)	16.6 (15.6, 17.7)	1355 (96)	18.2 (17.7, 18.8)	615.6 (598.2, 633.5)	519 (96)	19.6 (18.8, 20.4)
Yes	8 (2)	22.5 (14.6, 34.6)	510.4 (352.1, 740.0)	2 (1)	13.5 (7.2, 25.4)	62 (4)	14.9 (12.9, 17.3)	635.9 (555.3, 728.1)	19 (4)	22.6 (18.2, 28.0)
p_diff_		0.35	0.65		0.52		*0.009*	0.65		0.21
Age at menopause										
≤ 47 years						368 (33)	15.9 (15.0, 16.8)	644.4 (609.6, 681.3)	124 (30)	21.5 (19.8, 23.3)
48–51 years						406 (36)	17.4 (16.5, 18.4)	601.2 (570.7, 633.5)	157 (38)	19.9 (18.5, 21.4)
> 51 years						353 (31)	19.4 (18.3, 20.6)	660.1 (624.2, 698.1)	130 (32)	17.9 (16.5, 19.5)
p_trend_							*<0.0001*	0.81		*0.004*
Type of HT										
Never used HT						779 (75)	18.9 (17.8, 20.0)	618.3 (585.6, 653.0)	319 (81)	20.0 (18.7, 21.3)
Estrogen alone						81 (8)	13.6 (11.2, 16.5)	608.2 (507.6, 728.7)	20 (5)	18.9 (14.1, 25.3)
Estrogen + Progestin						177 (17)	16.2 (14.0, 18.8)	562.1 (488.7, 646.5)	54 (14)	16.7 (13.6, 20.4)
p_diff_, E alone vs. never							*0.005*	0.88		0.74
p_diff_, E + P vs. never							0.12	0.29		0.14
Ovulatory cycles^f^										
< = 368	143 (34)	27.2 (23.2, 31.9)	519.5 (456.0, 591.9)	56 (39)	17.3 (14.6, 20.6)	236 (22)	15.9 (14.8, 17.2)	654.8 (611.4, 701.4)	88 (21)	20.2 (18.2, 22.3)
369–414	120 (29)	26.8 (23.9, 30.2)	522.1 (474.3, 574.8)	49 (34)	15.7 (13.6, 18.0)	263 (24)	17.1 (15.9, 18.3)	598.9 (561.2, 639.2)	93 (23)	22.0 (20.0, 24.3)
415–450	80 (19)	28.7 (24.4, 33.7)	605.7 (530.3, 691.8)	26 (18)	19.1 (15.2, 24.1)	279 (25)	17.8 (16.6, 19.1)	618.9 (581.1, 659.2)	110 (27)	18.2 (16.6, 19.9)
> 450	73 (18)	29.9 (24.4, 36.6)	592.4 (501.1, 700.4)	14 (10)	13.6 (10.0, 18.6)	318 (29)	18.6 (17.5, 19.8)	613.8 (578.6, 651.1)	122 (30)	18.5 (17.0, 20.2)
p_trend_		0.57	0.27		0.45		*0.002*	0.25		0.06
BMI (kg/m^2^)										
< 18.5	9 (2)	28.8 (19.3, 43.1)	419.8 (298.7, 589.9)	1 (1)	15.9 (6.1, 41.2)	21 (2)	18.7 (14.6, 23.9)	478.1 (379.5, 602.4)	6 (1)	31.5 (21.5, 46.0)
18.5–24.99	267 (59)	27.1 (25.2, 29.1)	553.7 (520.7, 588.9)	94 (62)	16.2 (14.7, 17.8)	589 (43)	17.7 (16.9, 18.5)	603.9 (577.6, 631.4)	225 (44)	20.4 (19.1, 21.7)
25–29.99	131 (29)	27.4 (24.7, 30.4)	598.6 (548.0, 653.8)	43 (28)	17.9 (15.5, 20.8)	522 (39)	18.2 (17.3, 19.1)	624.8 (596.4, 654.6)	208 (41)	19.2 (18.0, 20.5)
≥30	46 (10)	26.2 (21.9, 31.2)	473.5 (406.8, 551.2)	14 (9)	16.8 (13.0, 21.7)	223 (16)	19.1 (17.7, 20.7)	682.5 (633.3, 735.6)	70 (14)	19.2 (17.1, 21.6)
p_trend_		0.75	0.60		0.54		0.14	*0.002*		0.10
Smoking										
Never	273 (57)	27.7 (25.8, 29.8)	543.1 (510.0, 578.4)	97 (56)	15.2 (14.0, 16.5)	817 (59)	18.8 (18.0, 19.5)	607.4 (585.0, 630.7)	319 (60)	18.2 (17.4, 19.2)
Former	114 (24)	28.5 (25.4, 32.0)	574.6 (520.2, 634.7)	39 (23)	15.6 (13.6, 17.8)	310 (22)	18.8 (17.6, 20.0)	660.1 (621.0, 701.7)	108 (20)	18.1 (16.6, 19.7)
Current	94 (20)	26.0 (22.9, 29.6)	575.2 (515.7, 641.7)	37 (21)	22.4 (19.4, 25.8)	266 (19)	15.7 (14.6, 16.8)	591.4 (553.4, 632.0)	102 (19)	27.1 (24.8, 29.6)
p_diff_, former vs. never		0.70	0.36		0.77		0.99	*0.03*		0.86
p_diff_, current vs. never		0.40	0.38		*<0.0001*		*<0.0001*	0.50		*<0.0001*

^a^2 controls missing CA125 and 9 missing CA153; ^b^ Restricted to 713 controls; ^c^Units: CA125: U/mL, CA15.3: mU/mL, HE4: pM; geometric means adjusted for matching factors, including study center (grouped by country), age at blood draw, fasting status, date and time of blood draw, menopausal status at blood draw, menstrual cycle phase for premenopausal women at blood draw, OC/HT use at blood draw, length of follow up. HT use and hysterectomy are each additionally adjusted for the other. *P* values based on continuous variables; ^d^p difference between pre- and postmenopausal: CA125 = <0.0001; CA15.3 = 0.02; HE4 = 0.92; ^e^ Trend among parous women; ^f^time between menarche and menopause with time subtracted for oral contraceptive use, pregnancy and breastfeeding; categories based on quartile cutpoints. Note: Italicized *p* values indicate statistically significant associations

Significant associations between epidemiologic factors and the investigated markers were predominantly observed among women who were postmenopausal at blood collection. Specifically, parity (*p* = 0.04), higher number of full-term pregnancies among parous women (p_trend_ = 0.02), older age at menopause (p_trend_ < 0.01), and greater estimated lifetime number of ovulatory cycles (p_trend_ < 0.01) were all associated with higher CA125 concentrations, whereas hysterectomy, unilateral oophorectomy, estrogen-only hormone therapy (vs. never use), and current smoking (vs. never smoking) were associated with lower concentrations (all associations *p* < 0.01). For CA125, no associations were observed among premenopausal women, with the exception of an inverse association between OC at blood collection and CA125 (users: 19 U/mL; non-users: 30 U/mL; *p* < 0.01). For CA15.3, higher BMI (p_trend_ < 0.01) and former smoking versus never smoking (*p* = 0.03) were associated with higher concentrations among postmenopausal women, while younger age at blood collection (*p* = 0.03) was associated with higher CA15.3 among premenopausal women. None of the remaining exposures were associated with circulating CA15.3. Older age at blood collection was associated with higher HE4 concentrations in postmenopausal women (p_trend_ < 0.01), whereas longer duration of OC use (p_trend_ < 0.01), higher parity (p_trend_ = 0.02), and older age at menopause were associated with lower concentrations. Current smoking, relative to never smoking, was associated with higher HE4 concentrations in both pre- and postmenopausal women (*p* < 0.01).

CA72.4 was evaluated as a dichotomous outcome (i.e., detectable vs. non-detectable concentrations), given than 82% of values were below the detection limit. We observed no associations between any of the examined epidemiologic risk factors and detectable vs. non-detectable CA72.4 concentrations, except suggestively higher CA72.4 with a higher BMI (≥25 vs. < 25, *p* = 0.05; Additional file 1: Table S1).

Tubal ligation (yes/no), age at menarche (<12, 12, 13, 14, 14+ years), IUD use (yes/no), and family history of breast cancer (yes/no) were not associated with any of the examined markers. The associations between oophorectomy, hysterectomy, ovulatory cycles with CA125, as well as the association between age at blood draw and CA15.3 in premenopausal women, were attenuated and no longer statistically significant after adjustment for the other investigated factors (i.e., adjusted for matching factors plus all significant correlates of the markers presented in the tables; Table 3 and Additional file 1: Table S2). The remaining associations were similar after adjustment. Finally, results were essentially unchanged in sensitivity analyses excluding eight outlying HE4 values.

**Table 3:** Multivariate adjusted associations between epidemiologic characteristics and CA125, CA15.3, and HE4 among controls postmenopausal at blood collection: EPIC^a^

	CA125		CA15.3		HE4^b^	
	beta	p	beta	p	beta	p
Age at blood draw (continuous)					0.016	*<0.0001*
Duration of oral contraceptive use (continuous)					−0.007	*0.009*
Parity (continuous)^c^	0.030	*0.03*			−0.036	*0.03*
Hysterectomy	−0.053	0.34				
Uniliateral oophorectomy	−0.104	0.18				
Age at menopause (continuous)	0.019	*0.0009*			−0.015	*0.03*
Type of HT (E alone vs. never)	−0.120	0.17				
Ovulatory cycles^c, d^(continuous)	−0.0001	0.76				
BMI (kg/m^2^; continuous)			0.013	*0.003*		
Smoking						
Former vs never	0.006	0.87	0.081	*0.03*	−0.003	0.95
Current vs never	−0.164	*<0.0001*	−0.018	0.66	0.385	*<0.0001*

^a^1 control missing CA125 and 8 missing CA153; ^b^ Restricted to 538 controls; ^c^model was run once including parity and excluding ovulatory cycles, then run again excluding parity and including ovulatory cycles. With the exception of ovulatory cycles, the betas and p-values shown are for the model including parity; ^d^ time between menarche and menopause with time subtracted for oral contraceptive use, pregnancy and breastfeeding. Note: Italicized *p* values indicate statistically significant associations

We observed few significant associations between the evaluated lifestyle and reproductive factors and the examined markers among ovarian cancer cases in the nested case-control study (Additional file 1: Table S3). There were no significant associations with CA125 among cases. However, among premenopausal women diagnosed with high-grade serous ovarian cancer over follow-up, current smoking was associated with lower CA125 (data not shown). Longer duration of OC use was associated with lower CA15.3 levels in postmenopausal women. Interestingly, higher parity and fewer ovulatory cycles were associated with lower premenopausal CA15.3 levels while the same exposures were associated with higher premenopausal HE4 levels. Among women diagnosed with high-grade serous disease, the association between OC use and HE4 levels persisted but the other associations did not (data not shown).

Finally, we investigated the discrimination of these markers before and after adjusting the markers (using biomarker residuals) for the epidemiologic factors identified as significant correlates in the cross-sectional analyses. These analyses were conducted in strata of time between blood collection and diagnosis (<1 year, 1 to <2 years, 2 to <3 years, ≥3 years). AUCs for the markers (individually and combined) were essentially unchanged when the marker values were adjusted for the epidemiologic correlates (e.g., AUC_<1 year_, postmenopausal women, markers unadjusted: 0.87 (95% confidence interval (CI): 0.81–0.93); marker residuals: 0.89 (95% CI: 0.84–0.95); Table 4).

**Table 4:** Discriminatory ability of ovarian cancer biomarkers adjusted for predictors of those biomarkers among women postmenopausal at blood collection: EPIC

	Time Between Blood Collection and Diagnosis			
	<1 year	1–2 years	2–3 years	>3 years
CA125				
*biomarker*	0.86 (0.79–0.92)	0.78 (0.71–0.84)	0.61 (0.51–0.70)	0.57 (0.54–0.61)
*adjusted biomarker* ^a^	0.85 (0.79–0.92)	0.78 (0.72–0.85)	0.60 (0.51–0.70)	0.57 (0.54–0.61)
CA15.3				
*biomarker*	0.66 (0.57–0.75)	0.60 (0.52–0.68)	0.61 (0.52–0.70)	0.56 (0.52–0.59)
*adjusted biomarker* ^a^	0.67 (0.57–0.76)	0.60 (0.52–0.68)	0.61 (0.52–0.70)	0.56 (0.52–0.59)
HE4				
*biomarker*	0.84 (0.77–0.90)	0.68 (0.61–0.76)	0.61 (0.52–0.70)	^b^
*adjusted biomarker* ^a^	0.84 (0.77–0.90)	0.70 (0.62–0.77)	0.62 (0.52–0.71)	^b^
CA72.4				
*biomarker*	0.76 (0.69–0.84)	0.67 (0.59–0.75)	0.61 (0.51–0.70)	^b^
*adjusted biomarker* ^a^	^c^	^c^	^c^	^b^
All biomarkers				CA125 and CA15.3^b^
*biomarker*	0.87 (0.81–0.93)	0.79 (0.73–0.85)	0.60 (0.51–0.70)	0.57 (0.54–0.61)
*adjusted biomarker* ^a^	0.89 (0.84–0.95)	0.80 (0.73–0.86)	0.62 (0.53–0.71)	0.57 (0.54–0.61)

^a^Biomarker residuals accounting for significant predictors: CA125: parity, hysterectomy, unilateral oophorectomy, age at menopause, E only HT, ovulatory cycles, current smoking; CA15.3: BMI, former smoking; HE4: age at blood draw, OC use, parity, age at menopause, current smoking; CA72.4: none; ^b^HE4 and CA72.4 only measured in cases diagnosed within 3 years of blood collection; ^c^No predictors identified

## Discussion

We present results from a large, cross-sectional study evaluating lifestyle and reproductive factors and ovarian cancer early detection markers. Adjustment for the identified correlates of these markers in early detection prediction models did not improve discrimination.

We confirmed previously reported observations [8, 13–16] of lower CA125 levels in post- vs. premenopausal women. CA15.3 levels were significantly higher among postmenopausal women. HE4 concentrations did not vary by menopausal status. We examined the effect of age at blood collection within strata of menopausal status and did not observe significant associations for CA125 and CA15.3, with the exception of a significant inverse association between age and CA15.3 among women who were premenopausal at blood collection. Large prior studies have reported a modest inverse association between age and CA125 [7, 8], evident in both pre- [8] and postmenopausal women [7, 8], whereas prior studies on CA15.3 observed a modest positive [16, 17] or no [23] association. However, neither of the studies observing a positive association between age and CA15.3 accounted for menopausal status at blood collection. We observed higher HE4 levels with older age only among women postmenopausal at blood collection. A positive association between age and HE4 has been previously reported (reviewed in [10, 24]). Older age at menopause was positively associated with CA125 concentrations, as has been observed previously [6–8], and inversely associated with HE4. We observed no association between age at menopause and CA15.3.

Among postmenopausal women, there was a modest inverse association between longer duration of OC use and HE4 concentrations. OC use has not previously been associated with circulating HE4 [11, 25]. However, data on OC duration are sparse. HE4 is expressed through the female reproductive tract [26], with the exception of the ovary. OC use inhibits cyclic proliferation leading to endometrial atrophy and predecidual changes in the stroma [27], though this is somewhat dependent on formulation. Therefore, OC use may impact HE4 concentrations via the effect on the endometrium. OC use may also impact mucin expression through upregulation of proinflammatory pathways recently shown to affect immunity in the distal reproductive tract [28]. Circulating concentrations of CA125 did not differ by duration of past OC use in our study, consistent with prior investigations [7, 23]. Use of estrogen-alone HT was associated with lower CA125 concentrations; these associations persisted in multivariable models but were only evident among women with hysterectomy in stratified models. Administered transdermal 17ß-estradiol has previously been associated with an increase in circulating CA125 in women without hysterectomy [29] and HT use (overall; formulation not specified) was associated with higher CA125 in the Prostate, Lung, Colorectal and Ovarian Cancer Screening Trial (PLCO) [6]. However, a positive association between HT and CA125 has not been universally observed [23, 30]. Cengiz et al. observed lower CA15.3 among women using estrogen-alone HT [31] in an analysis limited to women with hysterectomy and bilateral salpingo-oophorectomy. Thus, comparability to our study population is limited. One additional investigation observed no association between HT use (overall) and circulating CA15.3 [23]. HT use was not associated with HE4, consistent with others [12], or CA72.4 in the current investigation.

Parity was associated with higher CA125 concentrations and lower HE4 concentrations among women who were postmenopausal at blood collection. The endometrium is a major source of CA125 in healthy women, and it is plausible that the extensive pregnancy-induced changes in the endometrium contribute to long-term changes in circulating CA125 and HE4. Further, data suggest CA125 increases during early pregnancy and close to delivery [32–36]; it is plausible that the higher concentrations observed in pregnancy persist post-pregnancy. One study reported higher CA125 concentrations among women reporting parity of two or higher [37], however, an association between parity and CA125 has not consistently been observed among healthy women [7]. Prior data suggest lower CA125 concentrations in uterine flushing from women with recurrent miscarriages [38]. In turn, recurrent miscarriage is associated with lower parity. Additional studies are needed to clarify the impact of parity on subsequent circulating CA125 concentrations. The inverse association between parity and HE4 concentrations is consistent with one prior investigation [39]. However, other investigations have observed no association between parity and HE4 [11, 12]. We observed no association between parity and CA72.4 or CA15.3; for CA15.3, this is consistent with an earlier study [23].

Higher BMI was associated with higher CA15.3 concentrations among postmenopausal women in our study. This is consistent with a prior study [17] in men and women, suggesting that the effect is not explained solely by higher estrogen levels in obese postmenopausal women. An additional small study (*n* < 50) reported no significant association between BMI and circulating CA15.3 [40]. However, the analysis was limited to comparisons between obese vs. non-obese (BMI ≥30 vs. <30). We observed no association between BMI and CA125, HE4, or CA72.4. In the Prostate, Lung, Colorectal and Ovarian Cancer Screening Trial (PLCO), obese women had 3% lower CA125, relative to normal weight women (*p* < 0.001) [6]. Only 15% of the participants in the current investigation were obese (*n* = 271), as compared to almost 24% of PLCO study participants (*n* = 6063), and the current study was not statistically powered to detect such a small relative difference. Previous investigations on BMI and HE4 are mixed, reporting no [9, 10, 39], inverse [41], and positive [12] associations.

Current smoking was inversely associated with CA125 concentrations. This is consistent with three prior investigations in recent large, well-characterized populations [6–8], though significant associations were not observed in smaller prior studies [39, 42, 43]. Smoking may reduce CA125 concentrations via its effect on endogenous estrogens. Smoking is inversely associated with endogenous estrogens [44], whereas administered estradiol is associated with higher circulating CA125 [29]. Further, smoking is associated with earlier age at menopause [45], which, in turn, is associated with lower CA125. Finally, CA125 is expressed in the respiratory tract [46], and smoking may reduce circulating CA125 concentrations via damage to the respiratory tract epithelia or via more general immunosuppressive effects. We observed a higher concentrations of HE4 among women reporting current smoking, compared to never smokers, consistent with others (reviewed in [10]). As with CA125, HE4 is expressed in the oral cavity and respiratory tract, and it has been hypothesized that higher HE4 in smokers may be due to smoking-induced inflammation [41]. However, the mechanisms underlying the associations between CA125 and HE4 and smoking remain to be fully characterized. Former smoking, but not current smoking, was associated with higher CA15.3 concentrations in this investigation. One prior investigation observed no association between smoking and CA15.3 concentrations [17].

As expected, and consistent with prior studies [7, 47], we observed lower CA125 concentrations among women reporting hysterectomy, though this association did not persist in the fully adjusted model. A similar pattern was observed for oophorectomy. While ovarian cells do not express CA125, this marker is expressed in the fallopian tube epithelium. Prior studies have not observed an association between oophorectomy and CA125 [6, 7, 48] or have concluded that the decline in CA125 after bilateral salpingectomy-oophorectomy is not due to ovarian CA125 [47]. In our investigation, unilateral oophorectomy was associated with CA125 only before adjustment for hysterectomy and HT use, supporting prior observations that oophorectomy is not independently associated with CA125 concentrations. CA15.3, HE4, and CA72.4 were similar in women with and without reported hysterectomy or bilateral oophorectomy.

Significant associations observed in this study were predominantly observed among women who were postmenopausal at blood collection. CA125 and CA15.3 have been reported to vary across the menstrual cycle [20, 21]; substantial variation was not observed among premenopausal women in this investigation. However, we (i) adjusted for menstrual cycle phase and (ii) used phase-specific residuals to evaluate potential variability. Results were similar with both approaches. We were unable to assess cross-sectional associations during individual phases of the menstrual cycle. Our cross-sectional analysis between epidemiologic factors and early detection markers yielded few significant associations in women who subsequently developed ovarian cancer. Among high grade serous cases, significant findings were limited to only current smoking and lower CA125 in premenopausal women and oral contraceptives use with lower CA15.3 in postmenopausal women but may be due to chance given these associations were not observed in controls.

We hypothesized that inclusion of significant correlates of the evaluated early detection markers would improve discrimination of the early detection prediction models including these markers as this would, in part, account for sources of variation in these markers due to factors other than ovarian malignancy. However, we observed no improvement of the AUC in models adjusting for the epidemiologic correlates identified in the cross-sectional analyses and results from this study do not support the approach of adjusting CA125, CA15.3, HE4 or CA72.4 concentrations for their correlates to improve ovarian early detection models. An alternative approach would be to develop personalized cutpoints for the markers, based on a woman’s individual characteristics, as has been proposed for CA125 by menopausal status [8]. The current study was not designed to define or assess the utility of individualized cutpoints for ovarian cancer early detection; however, this should be explored in future studies designed for this purpose.

## Conclusions

This investigation adds to the limited data on correlates of CA125, CA15.3, HE4, and CA72.4 in healthy women, and provides the first data by menopausal status at blood collection. While we did not observe improvements in discrimination of early detection prediction models after accounting for these correlates, this data may inform future research on the development of individualized early detection marker cutpoints based on epidemiologic factors.

## Additional file

Additional file 1: Table S1. Association between epidemiologic characteristics and high CA72.4 by menopausal status at blood collection in healthy women: EPIC. **Table S2.** Multivariate adjusted association between epidemiologic characteristics and CA125, CA15.3 and HE4 by menopausal status at blood collection in healthy women: EPIC. **Table S3.** Association between epidemiologic characteristics and CA125, CA15.3, and HE4 by menopausal status at blood collection in women with ovarian cancer: EPIC (DOCX 75 kb)

## Abbreviations

AUC: Area under the receiver operating characteristic curve
BMI: Body mass index (kilograms/meter squared; kg/m^2^)
CA125: Cancer antigen 125, also known as mucin 16 (MUC16)
CA15.3: Cancer antigen 15.3, also known as mucin 1 (MUC1)
CA72.4: Cancer antigen 72.4
CI: Confidence interval
CV: Coefficient of variation
ECL: Electrochemiluminescence
EPIC: European Prospective Investigation into Cancer and Nutrition (EPIC)
HE4: Human epididymis protein 4
HT: Menopausal hormone therapy
IARC: International Agency for Research on Cancer
MSD: Meso Scale Discovery
OC: Oral contraceptive
PLCO: Prostate, Lung, Colorectal and Ovarian cancer screening trial
QC: Quality control
