# Supplementary material for: Correlates of circulating ovarian cancer early detection markers and their contribution to discrimination of early detection models: results from the EPIC cohort
Source: J Ovarian Res. 2017 Mar 20;10:20. doi: 10.1186/s13048-017-0315-6 (PMC5360038; doi:10.1186/s13048-017-0315-6)
Supplement: Additional file 1: Table S1. — Association between epidemiologic characteristics and high CA72.4 by menopausal status at blood collection in healthy women: EPIC. Table S2. Multivariate adjusted association between epidemiologic characteristics and CA125, CA15.3 and HE4 by menopausal status at blood collection in healthy women: EPIC. Table S3. Association between epidemiologic characteristics and CA125, CA15.3, and HE4 by menopausal status at blood collection in women with ovarian cancer: EPIC (DOCX 75 kb) [file 13048_2017_315_MOESM1_ESM.docx]

| **Table S1. Association between epidemiologic characteristics and high CA72.4 by menopausal status at blood collection in healthy women: EPIC** | | | | | | | | | |
| --- | --- | --- | --- | --- | --- | --- | --- | --- | --- |
|  | **Premenopausal** | | | |  | **Postmenopausal** | | | |
|  | **Low* N=151 N (%)** | **High* N=24 N (%)** | **Crude  OR (95% CI)** | **p** |  | **Low* N=457 N (%)** | **High* N=81 N (%)** | **Crude  OR (95% CI)** | **p** |
|  |  |  |  |  |  |  |  |  |  |
| **Age at blood draw** |  |  |  |  |  |  |  |  |  |
| ≤40 | 28 (19) | 5 (21) | 1.00 (ref) |  |  |  |  |  |  |
| >40 | 123 (81) | 19 (79) | 0.87 (0.30, 2.52) | 0.79 |  |  |  |  |  |
|  |  |  |  |  |  |  |  |  |  |
| ≤60 |  |  |  |  |  | 217 (47) | 42 (52) | 1.00 (ref) |  |
| >60 |  |  |  |  |  | 240 (53) | 39 (48) | 0.84 (0.52, 1.35) | 0.47 |
|  |  |  |  |  |  |  |  |  |  |
| **Oral contraceptive use** |  |  |  |  |  |  |  |  |  |
| Never | 40 (27) | 8 (36) | 1.00 (ref) |  |  | 251 (57) | 50 (63) | 1.00 (ref) |  |
| Ever | 108 (73) | 14 (64) | 0.65 (0.25, 1.66) | 0.37 |  | 193 (43) | 29 (37) | 0.75 (0.46, 1.24) | 0.26 |
|  |  |  |  |  |  |  |  |  |  |
| Never | 40 (28) | 8 (40) | 1.00 (ref) |  |  | 251 (59) | 50 (67) | 1.00 (ref) |  |
| ≥5 years | 58 (40) | 8 (40) | 0.69 (0.24, 1.99) | 0.49 |  | 86 (20) | 9 (12) | 0.53 (0.25, 1.11) | 0.09 |
| >5 years | 47 (32) | 4 (20) | 0.43 (0.12, 1.52) | 0.19 |  | 92 (21) | 16 (21) | 0.87 (0.47, 1.61) | 0.66 |
|  |  |  |  |  |  |  |  |  |  |
| **Parity** |  |  |  |  |  |  |  |  |  |
| Nulliparous | 14 (10) | 4 (20) | 1.00 (ref) |  |  | 54 (13) | 8 (11) | 1.00 (ref) |  |
| Parous | 132 (90) | 16 (80) | 0.42 (0.12, 1.45) | 0.17 |  | 375 (87) | 62 (89) | 1.12 (0.51, 2.46) | 0.79 |
|  |  |  |  |  |  |  |  |  |  |
| 0 | 14 (10) | 4 (20) | 1.00 (ref) |  |  | 54 (13) | 8 (12) | 1.00 (ref) |  |
| 1-2 | 82 (61) | 10 (50) | 0.43 (0.12, 1.55) | 0.20 |  | 224 (54) | 35 (51) | 1.06 (0.46, 2.40) | 0.90 |
| >2 | 38 (28) | 6 (30) | 0.55 (0.14, 2.25) | 0.41 |  | 138 (33) | 26 (38) | 1.27 (0.54, 2.98) | 0.58 |
|  |  |  |  |  |  |  |  |  |  |
| **Uniliateral oophorectomy** |  |  |  |  |  |  |  |  |  |
| No | 150 (99) | 23 (96) | 1.00 (ref) |  |  | 442 (97) | 77 (95) | 1.00 (ref) |  |
| Yes | 1 (1) | 1 (4) | 6.52 (0.39, 107.9) | 0.19 |  | 15 (3) | 4 (5) | 1.53 (0.50, 4.74) | 0.46 |
|  |  |  |  |  |  |  |  |  |  |
| **Hysterectomy** |  |  |  |  |  |  |  |  |  |
| No | 134 (98) | 20 (100) | -- |  |  | 313 (85) | 51 (91) | 1.00 (ref) |  |
| Yes | 3 (2) | 0 (0) | -- | -- |  | 55 (15) | 5 (9) | 0.56 (0.21, 1.46) | 0.23 |
|  |  |  |  |  |  |  |  |  |  |
| **Age at menopause** |  |  |  |  |  |  |  |  |  |
| <= 47 years |  |  |  |  |  | 114 (32) | 17 (27) | 1.00 (ref) |  |
| 48 - 51 years |  |  |  |  |  | 130 (37) | 28 (44) | 1.44 (0.75, 2.78) | 0.27 |
| > 51 years |  |  |  |  |  | 112 (31) | 18 (29) | 1.08 (0.53, 2.20) | 0.84 |
|  |  |  |  |  |  |  |  |  |  |
| **Hormone Replacement Therapy** | |  |  |  |  |  |  |  |  |
| Never used HRT |  |  |  |  |  | 307 (67) | 50 (62) | 1.00 (ref) |  |
| Ever used HRT |  |  |  |  |  | 150 (33) | 31 (38) | 1.27 (0.78, 2.07) | 0.34 |
|  |  |  |  |  |  |  |  |  |  |
| **Ovulatory cycles^¶^** |  |  |  |  |  |  |  |  |  |
| ≤ 368 | 42 (44) | 7 (64) | 1.00 (ref) |  |  |  |  |  |  |
| > 368 | 53 (56) | 4 (36) | 0.45 (0.12, 1.65) | 0.23 |  |  |  |  |  |
|  |  |  |  |  |  |  |  |  |  |
| ≤ 368 |  |  |  |  |  | 78 (22) | 10 (18) | 1.00 (ref) |  |
| 369-414 |  |  |  |  |  | 81 (23) | 12 (22) | 1.16 (0.47, 2.83) | 0.75 |
| 415-450 |  |  |  |  |  | 94 (26) | 16 (29) | 1.33 (0.57, 3.09) | 0.51 |
| > 450 |  |  |  |  |  | 105 (29) | 17 (31) | 1.26 (0.55, 2.91) | 0.58 |
|  |  |  |  |  |  |  |  |  |  |
| **BMI (kg/m^2^)** |  |  |  |  |  |  |  |  |  |
| <25 | 85 (66) | 10 (43) | 1.00 (ref) |  |  | 193 (44) | 38 (51) | 1.00 (ref) |  |
| ≥25 | 44 (34) | 13 (57) | 2.51 (1.02, 6.18) | 0.05 |  | 241 (56) | 37 (49) | 0.78 (0.48, 1.27) | 0.32 |
|  |  |  |  |  |  |  |  |  |  |
| **Smoking** |  |  |  |  |  |  |  |  |  |
| Never | 80 (54) | 17 (71) | 1.00 (ref) |  |  | 270 (60) | 49 (61) | 1.00 (ref) |  |
| Former | 36 (24) | 3 (13) | 0.39 (0.11, 1.42) | 0.15 |  | 99 (22) | 9 (11) | 0.50 (0.24, 1.06) | 0.07 |
| Current | 33 (22) | 4 (17) | 0.57 (0.18, 1.82) | 0.34 |  | 80 (18) | 22 (28) | 1.52 (0.86, 2.66) | 0.15 |
|  |  |  |  |  |  |  |  |  |  |
| *Low < 1.119 U/mL; High >1.119 U/mL  ^¶^time between menarche and menopause with time subtracted for oral contraceptive use, pregnancy and breastfeeding**;** categories based on quartile cutpoints; ‡CA72.4 is elevated (>1.119) in 13% of premenopausal controls and 15% of postmenopausal controls, p = 0.46 | | | | | | | | | |

| **Table S2. Multivariate adjusted association between epidemiologic characteristics and CA125 and CA15.3 by menopausal status at blood collection in healthy women: EPIC*** | | | | | | | | | | | |
| --- | --- | --- | --- | --- | --- | --- | --- | --- | --- | --- | --- |
|  | **PREMENOPAUSAL** | | | | |  | **POSTMENOPAUSAL** | | | | |
|  |  | **CA125 (U/mL)** | **CA15.3 (mU/mL)** | **HE4 (pM)** | |  |  | **CA125 (U/mL)** | **CA15.3 (U/mL)** | **HE4 (pM)** | |
|  | **N* (%)** | **Mean (95% CI)†** | **Mean (95% CI)†** | **N* (%)** | **Mean (95% CI)†** |  | **N* (%)** | **Mean (95% CI)†** | **Mean (95% CI)**† | **N* (%)** | **Mean (95% CI)†** |
| **Menopausal status‡** | 485 (26) | 28.1 (26.1, 30.3) | 566.8 (529.2, 607.0) | 175 (25) | 19.1 (17.3, 21.1) |  | 1417 (24) | 17.9 (17.3, 18.6) | 612.4 (592.8, 632.7) | 538 (75) | 18.8 (18.0, 19.6) |
|  |  |  |  |  |  |  |  |  |  |  |  |
| **Age at blood draw** |  |  |  |  |  |  |  |  |  |  |  |
| < 41 | 75 (15) | 27.1 (23.0, 31.9) | 600.7 (522.7, 690.3) | 33 (19) | 18.1 (14.8, 22.3) |  | 0 (0) | -- | -- | 0 (0) | -- |
| 41-50 | 269 (55) | 29.3 (27.1, 31.7) | 557.9 (521.6, 596.8) | 103 (59) | 17.0 (15.5, 18.6) |  | 47 (3) | 18.9 (16.0, 22.4) | 542.7 (462.1, 637.5) | 9 (2) | 16.4 (12.1, 22.3) |
| 51-60 | 141 (29) | 24.8 (21.9, 28.1) | 529.8 (475.8, 589.9) | 39 (22) | 14.3 (12.0, 17.1) |  | 697 (49) | 17.9 (17.2, 18.7) | 632.2 (606.0, 659.5) | 250 (46) | 18.6 (17.5, 19.7) |
| 61-70 | 0 (0) | -- | -- | 0 (0) | -- |  | 586 (41) | 17.8 (16.9, 18.6) | 611.2 (583.8, 639.8) | 227 (42) | 19.8 (18.6, 21.1) |
| > 70 | 0 (0) | -- | -- | 0 (0) | -- |  | 87 (6) | 20.9 (18.3, 23.9) | 572.8 (504.1, 650.8) | 52 (10) | 25.9 (22.3, 30.0) |
| p_trend_ |  | 0.36 | 0.20 |  | 0.11 |  |  | 0.44 | 0.49 |  | **0.0003** |
|  |  |  |  |  |  |  |  |  |  |  |  |
| **Age at menarche** |  |  |  |  |  |  |  |  |  |  |  |
| < 12 | 93 (20) | 27.8 (24.4, 31.7) | 542.1 (485.4, 605.4) | 33 (20) | 15.8 (13.5, 18.6) |  | 171 (13) | 17.9 (16.4, 19.5) | 622.5 (572.4, 676.9) | 63 (12) | 20.1 (17.9, 22.6) |
| 12 | 94 (20) | 27.7 (24.3, 31.6) | 574.3 (514.7, 640.9) | 33 (20) | 17.4 (14.9, 20.3) |  | 257 (19) | 18.0 (16.8, 19.3) | 593.2 (555.2, 633.8) | 102 (20) | 19.7 (18.0, 21.5) |
| 13 | 118 (26) | 26.4 (23.5, 29.6) | 559.6 (508.6, 615.9) | 48 (29) | 16.8 (14.8, 19.2) |  | 301 (22) | 17.8 (16.7, 19.0) | 629.4 (591.8, 669.4) | 117 (23) | 19.5 (18.0, 21.2) |
| 14 | 94 (20) | 28.1 (24.7, 32.1) | 510.4 (457.2, 569.7) | 34 (20) | 16.5 (14.1, 19.2) |  | 336 (25) | 18.6 (17.5, 19.8) | 617.0 (582.4, 653.7) | 129 (25) | 18.7 (17.3, 20.2) |
| > 14 | 60 (13) | 27.2 (23.1, 32.0) | 586.6 (511.2, 673.2) | 20 (12) | 15.9 (12.7, 19.9) |  | 300 (22) | 17.8 (16.7, 19.0) | 600.0 (563.8, 638.5) | 104 (20) | 20.3 (18.7, 22.2) |
| p_trend_ |  | 0.92 | 0.99 |  | 0.97 |  |  | 0.86 | 0.82 |  | 0.92 |
|  |  |  |  |  |  |  |  |  |  |  |  |
| **Oral contraceptive use** | |  |  |  |  |  |  |  |  |  |  |
| Never | 145 (31) | 27.9 (24.9, 31.2) | 509.3 (463.8, 559.2) | 48 (28) | 15.7 (13.6, 18.1) |  | 776 (56) | 18.3 (17.5, 19.1) | 626.9 (602.2, 652.7) | 301 (58) | 20.0 (19.0, 21.1) |
| Ever | 320 (69) | 27.4 (25.5, 29.4) | 571.6 (538.6, 606.5) | 122 (72) | 16.9 (15.6, 18.3) |  | 604 (44) | 17.9 (17.0, 18.8) | 597.6 (570.5, 626.1) | 222 (42) | 19.0 (17.9, 20.3) |
| p_diff_ |  | 0.81 | 0.06 |  | 0.41 |  |  | 0.54 | 0.15 |  | 0.26 |
| >= 2 years | 101 (22) | 27.3 (24.1, 31.1) | 596.8 (536.1, 664.3) | 44 (27) | 16.9 (14.7, 19.5) |  | 153 (12) | 18.8 (17.2, 20.6) | 607.5 (556.7, 662.9) | 51 (10) | 20.7 (18.3, 23.5) |
| > 2 - 5 years | 68 (15) | 30.0 (25.8, 34.9) | 527.7 (464.8, 599.1) | 22 (13) | 17.9 (14.9, 21.6) |  | 111 (8) | 18.0 (16.2, 20.0) | 574.3 (518.7, 635.8) | 44 (9) | 17.6 (15.4, 20.1) |
| > 5 - 10 years | 77 (17) | 26.0 (22.5, 30.1) | 571.1 (506.4, 644.1) | 27 (16) | 19.5 (16.4, 23.2) |  | 127 (10) | 17.0 (15.4, 18.8) | 598.4 (543.4, 658.9) | 46 (9) | 20.8 (18.1, 23.8) |
| > 10 years | 62 (14) | 26.7 (22.3, 32.0) | 617.1 (531.4, 716.5) | 24 (15) | 14.8 (11.8, 18.6) |  | 161 (12) | 17.9 (16.3, 19.6) | 616.1 (564.2, 672.8) | 62 (12) | 16.7 (14.8, 18.9) |
| p_trend_ |  | 0.57 | 0.13 |  | 0.56 |  |  | 0.56 | 0.86 |  | **0.01** |
| p_trend_^§^ |  | 0.71 | 0.18 |  | 0.36 |  |  | 0.78 | 0.52 |  | **0.03** |
|  |  |  |  |  |  |  |  |  |  |  |  |
| **Parity** |  |  |  |  |  |  |  |  |  |  |  |
| Nulliparous | 48 (11) | 29.9 (24.9, 35.8) | 576.8 (495.6, 671.3) | 18 (11) | 14.4 (11.6, 17.8) |  | 152 (12) | 16.5 (15.1, 18.1) | 607.0 (557.2, 661.2) | 62 (12) | 21.5 (19.3, 24.0) |
| Parous | 406 (89) | 27.4 (25.8, 29.1) | 548.4 (521.8, 576.4) | 148 (89) | 16.8 (15.7, 18.0) |  | 1163 (88) | 18.2 (17.6, 18.8) | 611.0 (592.5, 630.0) | 437 (88) | 19.3 (18.5, 20.1) |
| p_diff_ |  | 0.39 | 0.54 |  | 0.18 |  |  | 0.06 | 0.89 |  | 0.07 |
| 1 child | 74 (17) | 28.2 (24.3, 32.7) | 509.8 (452.0, 575.1) | 23 (15) | 15.8 (13.2, 18.9) |  | 200 (15) | 17.3 (16.0, 18.8) | 602.8 (558.7, 650.3) | 84 (17) | 19.6 (17.8, 21.5) |
| 2 children | 215 (50) | 27.6 (25.3, 30.0) | 557.7 (520.1, 598.1) | 69 (45) | 15.4 (13.8, 17.1) |  | 513 (40) | 18.2 (17.3, 19.1) | 597.2 (569.7, 625.9) | 175 (36) | 19.6 (18.3, 20.9) |
| 3 children | 75 (17) | 26.8 (23.2, 31.0) | 559.5 (496.9, 629.9) | 38 (25) | 18.7 (16.2, 21.5) |  | 270 (21) | 18.1 (16.9, 19.4) | 623.2 (584.4, 664.5) | 106 (22) | 19.2 (17.6, 20.9) |
| 4+ children | 22 (5) | 29.9 (22.7, 39.4) | 509.6 (406.9, 638.1) | 6 (4) | 23.4 (15.7, 34.9) |  | 157 (12) | 19.0 (17.3, 20.9) | 640.1 (586.5, 698.5) | 58 (12) | 18.0 (16.1, 20.3) |
| p_trend_ |  | 0.64 | 0.98 |  | **0.03** |  |  | **0.03** | 0.3 |  | **0.04** |
| p_trend_^§^ |  | 0.99 | 0.64 |  | 0.09 |  |  | 0.21 | 0.16 |  | 0.36 |
|  |  |  |  |  |  |  |  |  |  |  |  |
| **Hysterectomy** |  |  |  |  |  |  |  |  |  |  |  |
| No | 411 (97) | 27.5 (25.9, 29.2) | 549.4 (523.5, 576.5) | 154 (98) | 16.6 (15.6, 17.7) |  | 972 (86) | 17.8 (17.2, 18.5) | 594.5 (574.3, 615.4) | 364 (86) | 19.4 (18.5, 20.3) |
| Yes | 12 (3) | 20.4 (14.0, 29.7) | 649.1 (477.8, 881.8) | 3 (2) | 9.0 (4.9, 16.5) |  | 164 (14) | 16.8 (15.2, 18.6) | 572.1 (519.2, 630.5) | 60 (14) | 18.5 (16.1, 21.1) |
| p_diff_ |  | 0.13 | 0.29 |  | 0.05 |  |  | 0.31 | 0.49 |  | 0.52 |
|  |  |  |  |  |  |  |  |  |  |  |  |
| **Uniliateral oophorectomy** | |  |  |  |  |  |  |  |  |  |  |
| No | 477 (98) | 27.7 (26.2, 29.2) | 556.5 (531.2, 583.0) | 173 (99) | 16.6 (15.7, 17.7) |  | 1355 (96) | 18.2 (17.6, 18.7) | 616.0 (598.7, 633.9) | 519 (96) | 19.6 (18.9, 20.3) |
| Yes | 8 (2) | 22.6 (14.6, 35.1) | 526.2 (361.2, 766.7) | 2 (1) | 11.7 (6.4, 21.7) |  | 62 (4) | 16.1 (13.9, 18.7) | 626.9 (543.9, 722.6) | 19 (4) | 21.7 (17.6, 26.7) |
| p_diff_ |  | 0.37 | 0.77 |  | 0.27 |  |  | 0.13 | 0.81 |  | 0.35 |
|  |  |  |  |  |  |  |  |  |  |  |  |
| **Age at menopause** |  |  |  |  |  |  |  |  |  |  |  |
| <= 47 years |  |  |  |  |  |  | 341 (31) | 16.1 (15.2, 17.2) | 640.5 (603.9, 679.3) | 124 (30) | 21.1 (19.4, 22.9) |
| 48 - 51 years |  |  |  |  |  |  | 397 (36) | 17.4 (16.5, 18.4) | 606.5 (575.2, 639.5) | 157 (38) | 19.7 (18.3, 21.2) |
| > 51 years |  |  |  |  |  |  | 351 (32) | 19.0 (17.9, 20.2) | 657.7 (621.0, 696.6) | 130 (32) | 18.5 (17.1, 20.1) |
| p_trend_ |  |  |  |  |  |  |  | **0.0005** | 0.69 |  | **0.04** |
|  |  |  |  |  |  |  |  |  |  |  |  |
| **Type of HRT** |  |  |  |  |  |  |  |  |  |  |  |
| Never used HRT |  |  |  |  |  |  | 779 (75) | 18.6 (17.6, 19.7) | 618.4 (585.1, 653.6) | 319 (81) | 20.1 (18.8, 21.4) |
| Estrogen alone |  |  |  |  |  |  | 81 (8) | 14.9 (12.3, 18.1) | 603.9 (501.3, 727.6) | 20 (5) | 19.2 (14.3, 25.7) |
| Estrogen + Progestin |  |  |  |  |  |  | 177 (17) | 16.6 (14.3, 19.3) | 563.5 (488.8, 649.7) | 54 (14) | 16.0 (13.1, 19.5) |
| p_diff_, E alone vs. never | |  |  |  |  |  |  | **0.04** | 0.83 |  | 0.76 |
| p_diff_, E+P vs. never | |  |  |  |  |  |  | 0.25 | 0.31 |  | 0.06 |
|  |  |  |  |  |  |  |  |  |  |  |  |
| **Ovulatory cycles^¶^** | |  |  |  |  |  |  |  |  |  |  |
| <= 368 | 143 (34) | 27.1 (23.1, 31.9) | 520.5 (456.0, 594.2) | 56 (39) | 18.4 (15.4, 21.8) |  | 236 (22) | 17.7 (16.1, 19.5) | 672.0 (614.3, 735.1) | 88 (21) | 19.5 (17.1, 22.3) |
| 369-414 | 120 (29) | 26.8 (23.8, 30.2) | 519.0 (471.1, 571.8) | 49 (34) | 15.2 (13.2, 17.4) |  | 263 (24) | 17.7 (16.4, 19.0) | 613.8 (573.4, 657.1) | 93 (23) | 21.8 (19.7, 24.1) |
| 415-450 | 80 (19) | 28.8 (24.5, 33.8) | 605.3 (529.5, 692.0) | 26 (18) | 18.0 (14.3, 22.6) |  | 279 (25) | 17.5 (16.3, 18.8) | 624.6 (584.1, 668.0) | 110 (27) | 17.9 (16.3, 19.7) |
| > 450 | 73 (18) | 30.0 (24.4, 36.8) | 596.3 (503.3, 706.5) | 14 (10) | 13.5 (9.9, 18.5) |  | 318 (29) | 17.0 (15.7, 18.3) | 585.3 (544.2, 629.5) | 122 (30) | 19.4 (17.5, 21.5) |
| p_trend_ |  | 0.54 | 0.26 |  | 0.20 |  |  | 0.54 | 0.05 |  | 0.87 |
|  |  |  |  |  |  |  |  |  |  |  |  |
| **BMI (kg/m^2^)** |  |  |  |  |  |  |  |  |  |  |  |
| <18.5 | 9 (2) | 27.6 (18.3, 41.5) | 433.4 (306.6, 612.6) | 1 (1) | 16.5 (6.6, 41.6) |  | 21 (2) | 21.0 (16.4, 26.8) | 483.1 (382.2, 610.7) | 6 (1) | 25.5 (17.5, 37.0) |
| 18.5-24.99 | 267 (59) | 27.0 (25.1, 29.1) | 552.5 (519.1, 588.0) | 94 (62) | 16.2 (14.8, 17.7) |  | 589 (43) | 17.6 (16.8, 18.5) | 608.3 (581.6, 636.3) | 225 (44) | 20.3 (19.2, 21.6) |
| 25 - 29.99 | 131 (29) | 27.6 (24.8, 30.7) | 599.0 (547.6, 655.3) | 43 (28) | 18.1 (15.8, 20.8) |  | 522 (39) | 18.2 (17.3, 19.1) | 622.4 (594.0, 652.2) | 208 (41) | 19.2 (18.1, 20.4) |
| >=30 | 46 (10) | 26.1 (21.9, 31.3) | 475.7 (408.2, 554.2) | 14 (9) | 16.0 (12.6, 20.4) |  | 223 (16) | 19.1 (17.6, 20.6) | 674.8 (625.6, 727.7) | 70 (14) | 19.8 (17.7, 22.1) |
| p_trend_ |  | 0.84 | 0.59 |  | 0.70 |  |  | 0.24 | **0.008** |  | 0.31 |
|  |  |  |  |  |  |  |  |  |  |  |  |
| **Smoking** |  |  |  |  |  |  |  |  |  |  |  |
| Never | 273 (57) | 27.9 (25.9, 30.0) | 543.6 (510.1, 579.3) | 97 (56) | 15.2 (13.9, 16.6) |  | 817 (59) | 18.7 (18.0, 19.4) | 606.1 (583.7, 629.4) | 319 (60) | 18.2 (17.3, 19.1) |
| Former | 114 (24) | 28.0 (24.9, 31.5) | 582.0 (526.0, 643.8) | 39 (23) | 15.5 (13.5, 17.8) |  | 310 (22) | 18.8 (17.7, 20.1) | 659.7 (620.3, 701.6) | 108 (20) | 18.4 (16.9, 20.0) |
| Current | 94 (20) | 26.3 (23.1, 30.0) | 565.0 (505.7, 631.4) | 37 (21) | 22.5 (19.5, 26.0) |  | 266 (19) | 15.9 (14.8, 17.0) | 595.6 (557.1, 636.8) | 102 (19) | 26.8 (24.5, 29.3) |
| p_diff_, former vs. never | | 0.94 | 0.28 |  | 0.80 |  |  | 0.82 | **0.02** |  | 0.83 |
| p_diff_, current vs. never | | 0.46 | 0.56 |  | **<0.0001** |  |  | **0.0001** | 0.66 |  | **<0.0001** |
|  |  |  |  |  |  |  |  |  |  |  |  |
| **Packyears (among current smokers)** | | |  |  |  |  |  |  |  |  |  |
| <=11 | 34 (37) | 31.5 (23.9, 41.6) | 621.5 (515.0, 750.1) | 11 (31) | 23.2 (6.4, 84.8) |  | 53 (21) | 19.6 (16.6, 23.2) | 564.9 (485.1, 657.7) | 24 (25) | 24.1 (19.3, 30.0) |
| 12-19 | 21 (23) | 24.9 (17.9, 34.8) | 547.7 (437.0, 686.4) | 9 (25) | 13.3 (3.1, 57.4) |  | 67 (26) | 13.2 (11.3, 15.4) | 630.2 (547.7, 725.2) | 23 (24) | 26.3 (20.8, 33.3) |
| 20-31 | 26 (28) | 21.8 (15.8, 29.9) | 542.6 (437.7, 672.6) | 11 (31) | 34.3 (8.9, 132.2) |  | 68 (26) | 18.3 (15.9, 21.2) | 600.0 (526.0, 684.5) | 26 (27) | 28.9 (23.3, 35.8) |
| >31 | 12 (13) | 30.8 (19.2, 49.5) | 549.7 (398.9, 757.4) | 5 (14) | 18.5 (4.3, 79.5) |  | 69 (27) | 15.5 (13.3, 18.0) | 597.1 (520.4, 685.2) | 24 (25) | 28.4 (22.9, 35.2) |
| p_trend_ |  | 0.55 | 0.61 |  | 0.85 |  |  | 0.38 | 0.75 |  | 0.28 |
| *Includes 1 control missing CA125 and 8 missing CA153; ** Restricted to 713 controls; †Geometric means adjusted for BMI, parity, OC use, hysterectomy, and smoking status, plus the matching factors, including study center (grouped by country), age at blood draw, fasting status, date of blood draw, menstrual cycle phase for premenopausal women at blood, OC/HRT use at blood, length of follow up. Models in postmenopausal women are additionally adjusted for oophorectomy, age at menopause, and type of HRT. P values based on continuous variables; ‡p difference between pre- and postmenopausal: CA125: <0.0001; CA15.3: 0.08; HE4: 0.78; ^§^ Trend among parous women; ^¶^time between menarche and menopause with time subtracted for oral contraceptive use, pregnancy and breastfeeding**;** categories based on quartile cutpoints | | | | | | | | | | | |

| **Table S3. Association between epidemiologic characteristics and CA125, CA15.3, and HE4 by menopausal status at blood collection in women with ovarian cancer: EPIC*** | | | | | | | | | | | |
| --- | --- | --- | --- | --- | --- | --- | --- | --- | --- | --- | --- |
|  | **PREMENOPAUSAL** | | | | |  | **POSTMENOPAUSAL** | | | | |
|  |  | **CA125 (U/mL)** | **CA15.3 (mU/mL)** | **HE4 (pM)** | |  |  | **CA125 (U/mL)** | **CA15.3 (mU/mL)** | **HE4 (pM)** | |
|  | **N* (%)** | **Mean (95% CI)†** | **Mean (95% CI)†** | **N** (%)** | **Mean (95% CI)†** |  | **N* (%)** | **Mean (95% CI)†** | **Mean (95% CI)†** | **N* (%)** | **Mean (95% CI)†** |
| **Menopausal status‡** | 201 (25) | 34.9 (28.9, 42.1) | 569.3 (506.5, 640.0) | 46 (24) | 27.4 (19.5, 38.4) |  | 590 (75) | 25.5 (23.3, 27.9) | 644.2 (609.3, 681.2) | 147 (76) | 29.6 (25.6, 34.2) |
|  |  |  |  |  |  |  |  |  |  |  |  |
| **Age at blood draw** |  |  |  |  |  |  |  |  |  |  |  |
| < 41 | 30 (15) | 30.8 (21.5, 44.3) | 639.7 (511.0, 800.7) | 8 (17) | 19.9 (9.8, 40.7) |  | -- | -- | -- | -- | -- |
| 41-50 | 109 (54) | 35.9 (30.1, 42.9) | 538.1 (482.3, 600.5) | 27 (59) | 25.2 (18.6, 34.3) |  | 20 (3) | 27.6 (17.7, 43.1) | 420.7 (319.8, 553.3) | 2 (1) | 18.5 (5.8, 58.8) |
| 51-60 | 62 (31) | 35.5 (27.2, 46.3) | 585.8 (496.6, 691.0) | 11 (24) | 20.7 (11.0, 39.1) |  | 296 (50) | 26.2 (23.3, 29.4) | 678.6 (631.6, 729.1) | 70 (48) | 28.1 (23.0, 34.4) |
| 61-70 | -- | -- | -- | -- | -- |  | 241 (41) | 24.2 (21.3, 27.5) | 642.8 (594.2, 695.5) | 61 (41) | 34.9 (28.2, 43.1) |
| > 70 | -- | -- | -- | -- | -- |  | 33 (6) | 27.6 (19.1, 39.9) | 547.1 (435.8, 686.8) | 14 (10) | 35.0 (21.4, 57.1) |
| p_trend_ |  | 0.61 | 0.67 |  | 0.93 |  |  | 0.60 | 0.90 |  | 0.16 |
|  |  |  |  |  |  |  |  |  |  |  |  |
| **Age at menarche** | |  |  |  |  |  |  |  |  |  |  |
| < 12 | 34 (18) | 34.5 (24.9, 47.6) | 536.2 (439.7, 654.0) | 11 (25) | 25.6 (15.4, 42.5) |  | 70 (12) | 25.2 (19.9, 31.8) | 653.0 (564.1, 755.9) | 17 (12) | 32.4 (22.0, 47.8) |
| 12 | 45 (24) | 34.4 (26.2, 45.1) | 540.3 (457.4, 638.1) | 7 (16) | 17.3 (10.7, 28.0) |  | 85 (15) | 23.1 (18.7, 28.5) | 567.1 (497.0, 647.2) | 19 (14) | 28.2 (19.6, 40.5) |
| 13 | 60 (31) | 34.6 (27.5, 43.6) | 573.4 (497.5, 660.8) | 15 (34) | 15.6 (10.6, 22.9) |  | 127 (22) | 24.6 (20.8, 29.2) | 663.9 (596.6, 738.8) | 27 (20) | 26.4 (19.5, 35.9) |
| 14 | 28 (15) | 29.9 (20.9, 42.6) | 646.3 (519.8, 803.5) | 6 (14) | 19.3 (9.8, 38.0) |  | 143 (25) | 26.7 (22.7, 31.5) | 661.4 (596.8, 732.9) | 38 (28) | 30.8 (23.8, 39.7) |
| > 14 | 24 (13) | 40.7 (27.8, 59.8) | 510.9 (403.8, 646.4) | 5 (11) | 83.2 (36.9, 187.6) |  | 141 (25) | 25.2 (21.4, 29.6) | 644.0 (581.4, 713.3) | 37 (27) | 32.5 (24.9, 42.4) |
| p_trend_ |  | 0.80 | 0.69 |  | 0.48 |  |  | 0.61 | 0.50 |  | 0.73 |
|  |  |  |  |  |  |  |  |  |  |  |  |
| **Oral contraceptive use** | | |  |  |  |  |  |  |  |  |  |
| Never | 80 (42) | 36.0 (29.0, 44.8) | 551.9 (482.7, 631.1) | 20 (45) | 21.7 (13.9, 33.7) |  | 349 (61) | 25.6 (23.0, 28.5) | 648.4 (606.2, 693.4) | 87 (62) | 31.6 (26.5, 37.8) |
| Ever | 111 (58) | 33.4 (27.9, 40.0) | 566.6 (507.4, 632.7) | 24 (55) | 22.9 (15.6, 33.7) |  | 224 (39) | 24.9 (21.7, 28.6) | 625.0 (573.6, 681.1) | 53 (38) | 30.3 (23.9, 38.5) |
| p_diff_ |  | 0.63 | 0.79 |  | 0.88 |  |  | 0.79 | 0.53 |  | 0.80 |
| >= 2 years | 42 (22) | 40.7 (30.8, 53.8) | 546.6 (460.8, 648.4) | 9 (21) | 28.7 (15.0, 55.0) |  | 65 (12) | 21.5 (16.8, 27.4) | 621.2 (534.1, 722.5) | 13 (10) | 23.0 (14.5, 36.3) |
| > 2 -5 years | 24 (13) | 24.4 (16.8, 35.4) | 555.8 (442.6, 698.0) | 5 (12) | 19.3 (6.4, 58.3) |  | 49 (9) | 28.0 (21.2, 37.1) | 704.9 (593.2, 837.6) | 12 (9) | 36.1 (22.6, 57.7) |
| > 5 -10 years | 24 (13) | 37.6 (25.1, 56.4) | 536.4 (418.8, 686.9) | 4 (9) | 19.6 (6.2, 62.2) |  | 55 (10) | 25.9 (19.9, 33.7) | 633.0 (537.6, 745.3) | 12 (9) | 31.2 (19.4, 50.1) |
| > 10 years | 17 (9) | 27.4 (16.9, 44.4) | 677.4 (504.5, 909.4) | 5 (12) | 19.1 (6.5, 56.3) |  | 40 (7) | 21.3 (15.4, 29.5) | 448.3 (367.2, 547.2) | 9 (7) | 29.6 (16.7, 52.2) |
| p_trend_ |  | 0.24 | 0.26 |  | 0.69 |  |  | 0.47 | **0.001** |  | 0.87 |
| p_trend_^§^ |  | 0.41 | 0.27 |  | 0.05 |  |  | 0.95 | **0.0008** |  | 0.58 |
|  |  |  |  |  |  |  |  |  |  |  |  |
| **Parity** |  |  |  |  |  |  |  |  |  |  |  |
| Nulliparous | 40 (21) | 31.5 (23.7, 42.0) | 611.8 (513.0, 729.6) | 14 (32) | 12.7 (8.6, 18.6) |  | 87 (16) | 29.4 (23.9, 36.2) | 595.3 (523.2, 677.4) | 25 (18) | 33.1 (24.4, 45.1) |
| Parous | 150 (79) | 35.3 (30.6, 40.7) | 549.1 (502.9, 599.6) | 30 (68) | 29.1 (23.1, 36.7) |  | 460 (84) | 24.6 (22.5, 26.9) | 645.3 (610.3, 682.3) | 112 (82) | 30.0 (26.1, 34.5) |
| p_diff_ |  | 0.49 | 0.29 |  | **0.005** |  |  | 0.12 | 0.26 |  | 0.57 |
| 1 child | 35 (19) | 32.4 (23.7, 44.4) | 650.3 (539.3, 784.0) | 6 (15) | 29.0 (12.6, 67.2) |  | 77 (14) | 23.9 (19.1, 29.9) | 629.9 (547.3, 725.0) | 25 (19) | 28.5 (20.7, 39.2) |
| 2 children | 76 (41) | 35.9 (29.0, 44.4) | 516.8 (455.3, 586.7) | 11 (27) | 25.9 (16.7, 40.1) |  | 216 (40) | 25.4 (22.2, 28.9) | 628.6 (578.4, 683.1) | 47 (35) | 32.8 (26.2, 41.2) |
| 3 children | 20 (11) | 35.1 (22.9, 53.7) | 466.4 (362.0, 601.0) | 6 (15) | 28.2 (12.3, 64.7) |  | 99 (18) | 23.3 (19.1, 28.3) | 687.9 (608.4, 777.8) | 24 (18) | 31.2 (22.6, 43.0) |
| 4+ children | 13 (7) | 42.1 (24.7, 71.8) | 552.2 (402.1, 758.4) | 4 (10) | 42.1 (18.9, 93.6) |  | 59 (11) | 25.8 (19.9, 33.5) | 664.2 (564.3, 781.9) | 13 (10) | 27.9 (17.5, 44.4) |
| p_trend_ |  | 0.39 | **0.04** |  | **0.006** |  |  | 0.30 | 0.14 |  | 0.79 |
| p_trend_^§^ |  | 0.34 | 0.18 |  | 0.86 |  |  | 0.86 | 0.33 |  | 0.78 |
|  |  |  |  |  |  |  |  |  |  |  |  |
| **Hysterectomy** |  |  |  |  |  |  |  |  |  |  |  |
| No | 172 (98) | 34.3 (30.0, 39.1) | 555.9 (513.1, 602.3) | 41 (98) | 22.9 (18.4, 28.5) |  | 412 (86) | 24.4 (22.2, 26.8) | 630.9 (595.1, 668.9) | 107 (91) | 28.5 (24.7, 32.9) |
| Yes | 4 (2) | 23.2 (8.5, 63.6) | 611.5 (332.6, 1124.4) | 1 (2) | 15.0 (2.5, 88.9) |  | 65 (14) | 30.3 (23.0, 39.8) | 665.2 (563.5, 785.2) | 10 (9) | 42.9 (23.1, 79.7) |
| p_diff_ |  | 0.46 | 0.76 |  | 0.65 |  |  | 0.16 | 0.57 |  | 0.23 |
|  |  |  |  |  |  |  |  |  |  |  |  |
| **Uniliateral oophorectomy** | |  |  |  |  |  |  |  |  |  |  |
| No | 197 (98) | 35.2 (31.2, 39.8) | 571.6 (529.7, 616.7) | 46 (100) | -- |  | 575 (97) | 25.5 (23.5, 27.6) | 646.5 (615.0, 679.6) | 144 (98) | 30.9 (27.3, 35.0) |
| Yes | 4 (2) | 24.6 (9.6, 63.0) | 376.4 (210.0, 674.6) | 0 (0) | -- |  | 15 (3) | 23.9 (14.5, 39.6) | 596.5 (435.9, 816.3) | 3 (2) | 52.0 (20.4, 132.5) |
| p_diff_ |  | 0.46 | 0.17 |  |  |  |  | 0.81 | 0.62 |  | 0.28 |
|  |  |  |  |  |  |  |  |  |  |  |  |
| **Age at menopause** |  |  |  |  |  |  |  |  |  |  |  |
| ≤ 47 years |  |  |  |  |  |  | 111 (25) | 26.0 (21.5, 31.3) | 655.9 (583.0, 737.8) | 21 (18) | 30.7 (22.2, 42.5) |
| 48 - 51 years |  |  |  |  |  |  | 175 (39) | 23.2 (20.0, 27.0) | 683.9 (623.1, 750.7) | 37 (32) | 39.6 (30.6, 51.1) |
| > 51 years |  |  |  |  |  |  | 166 (37) | 24.8 (21.3, 28.9) | 609.2 (553.6, 670.4) | 57 (50) | 26.8 (21.9, 32.8) |
| p_trend_ |  |  |  |  |  |  |  | 0.69 | 0.37 |  | 0.41 |
|  |  |  |  |  |  |  |  |  |  |  |  |
| **Type of HRT** |  |  |  |  |  |  |  |  |  |  |  |
| Never used HRT |  |  |  |  |  |  | 330 (75) | 27.1 (23.1, 31.8) | 646.9 (583.8, 716.9) | 85 (79) | 33.6 (26.0, 43.5) |
| Estrogen alone |  |  |  |  |  |  | 36 (8) | 16.5 (9.7, 28.0) | 683.9 (487.7, 959.0) | 5 (5) | 16.0 (4.5, 57.0) |
| Estrogen + Progestin |  |  |  |  |  |  | 74 (17) | 24.5 (16.0, 37.4) | 579.5 (442.5, 759.0) | 18 (17) | 29.2 (12.8, 66.3) |
| p_diff_, E alone vs. never |  |  |  |  |  |  |  | 0.13 | 0.79 |  | 0.30 |
| p_diff_, E+P vs. never |  |  |  |  |  |  |  | 0.71 | 0.53 |  | 0.78 |
|  |  |  |  |  |  |  |  |  |  |  |  |
| **Ovulatory cycles^¶^** |  |  |  |  |  |  |  |  |  |  |  |
| <= 368 | 57 (32) | 39.7 (27.0, 58.5) | 463.2 (369.3, 580.9) | 12 (31) | 54.9 (31.2, 96.6) |  | 76 (16) | 26.2 (20.9, 32.9) | 656.6 (568.4, 758.5) | 19 (16) | 33.6 (23.0, 49.3) |
| 369-414 | 44 (25) | 37.8 (28.3, 50.5) | 490.1 (413.6, 580.8) | 13 (33) | 25.2 (16.1, 39.5) |  | 125 (27) | 21.7 (18.2, 25.9) | 673.7 (602.8, 753.0) | 25 (21) | 32.4 (23.3, 45.2) |
| 415-450 | 50 (28) | 31.5 (22.9, 43.2) | 638.7 (530.2, 769.4) | 9 (23) | 14.6 (8.2, 25.7) |  | 121 (26) | 28.7 (24.0, 34.4) | 660.9 (590.2, 739.9) | 28 (24) | 30.0 (22.2, 40.6) |
| > 450 | 28 (16) | 29.4 (18.2, 47.7) | 732.8 (552.3, 972.2) | 5 (13) | 5.5 (2.5, 12.3) |  | 140 (30) | 23.9 (20.2, 28.2) | 587.3 (528.9, 652.1) | 45 (38) | 29.7 (23.4, 37.8) |
| p_trend_ |  | 0.43 | **0.04** |  | **0.003** |  |  | 0.99 | 0.15 |  | 0.56 |
|  |  |  |  |  |  |  |  |  |  |  |  |
| **BMI (kg/m^2^)** |  |  |  |  |  |  |  |  |  |  |  |
| <18.5 | 1 (1) | 20.4 (3.1, 135.6) | 543.7 (163.8, 1804.9) | 0 (0) |  |  | 4 (1) | 10.9 (4.1, 29.4) | 630.9 (338.0, 1177.5) | 2 (1) | 57.9 (17.8, 188.4) |
| 18.5-24.99 | 111 (58) | 33.5 (28.3, 39.7) | 564.5 (506.9, 628.6) | 23 (58) | 22.4 (15.8, 31.8) |  | 252 (45) | 25.0 (22.1, 28.3) | 637.4 (588.9, 689.8) | 61 (44) | 30.0 (24.5, 36.8) |
| 25 - 29.99 | 54 (28) | 30.9 (24.2, 39.5) | 554.7 (475.0, 647.8) | 11 (28) | 15.6 (9.9, 24.6) |  | 193 (34) | 25.5 (22.1, 29.3) | 651.8 (596.3, 712.4) | 48 (34) | 32.4 (25.7, 40.8) |
| >=30 | 27 (14) | 50.1 (35.0, 71.7) | 637.5 (507.9, 800.1) | 6 (15) | 61.8 (26.1, 146.2) |  | 114 (20) | 25.9 (21.5, 31.2) | 680.4 (604.8, 765.5) | 29 (21) | 28.9 (21.1, 39.7) |
| p_trend_ |  | 0.11 | 0.44 |  | 0.26 |  |  | 0.58 | 0.38 |  | 0.74 |
|  |  |  |  |  |  |  |  |  |  |  |  |
| **Smoking** |  |  |  |  |  |  |  |  |  |  |  |
| Never | 101 (51) | 38.5 (32.4, 45.9) | 541.5 (485.3, 604.3) | 25 (56) | 24.7 (17.8, 34.4) |  | 323 (56) | 26.5 (23.8, 29.6) | 642.2 (599.6, 687.9) | 92 (64) | 32.3 (27.4, 38.0) |
| Former | 52 (26) | 35.1 (27.4, 45.0) | 590.2 (504.9, 689.9) | 8 (18) | 17.9 (9.2, 34.7) |  | 128 (22) | 26.3 (22.1, 31.3) | 613.7 (549.6, 685.2) | 26 (18) | 26.4 (19.2, 36.4) |
| Current | 47 (24) | 28.4 (21.8, 37.1) | 595.8 (504.0, 704.2) | 12 (27) | 24.8 (14.4, 42.7) |  | 126 (22) | 22.6 (19.0, 27.0) | 680.4 (608.5, 760.8) | 25 (17) | 33.8 (24.3, 47.1) |
| p_diff_, former vs. never |  | 0.56 | 0.38 |  | 0.43 |  |  | 0.92 | 0.50 |  | 0.29 |
| p_diff_, current vs. never |  | 0.07 | 0.36 |  | 0.99 |  |  | 0.14 | 0.40 |  | 0.81 |
|  |  |  |  |  |  |  |  |  |  |  |  |
| **Packyears (among current smokers)** | | |  |  |  |  |  |  |  |  |  |
| <=11 packyears | 15 (32) | 30.5 (15.8, 58.9) | 536.6 (373.5, 770.8) | 4 (33) | 23.0 (11.1, 47.6) |  | 26 (21) | 19.0 (13.4, 26.8) | 611.0 (486.5, 767.5) | 6 (24) | 27.3 (9.2, 81.0) |
| 12-19 | 15 (32) | 54.1 (29.4, 99.5) | 561.6 (401.3, 785.9) | 4 (33) | 23.9 (10.7, 53.3) |  | 27 (22) | 34.2 (24.1, 48.6) | 724.6 (575.0, 913.1) | 3 (12) | 39.4 (7.2, 216.1) |
| 20-31 | 12 (26) | 17.6 (9.1, 34.2) | 745.9 (517.2, 1075.5) | 4 (33) | 42.5 (18.7, 97.0) |  | 31 (25) | 18.4 (13.4, 25.2) | 703.3 (569.0, 869.4) | 6 (24) | 28.4 (10.7, 75.2) |
| >31 | 5 (11) | 16.7 (5.5, 50.6) | 850.2 (461.6, 1566.0) | 0 (0) |  |  | 38 (31) | 19.8 (14.8, 26.6) | 721.8 (594.3, 876.7) | 10 (40) | 48.0 (22.5, 102.6) |
| p_trend_ |  | 0.24 | 0.62 |  | 0.46 |  |  | 0.72 | 0.40 |  | 0.21 |
| *Includes 1 control missing CA125 and 8 missing CA153; ** Restricted to 193 cases; †geometric means adjusted for matching factors, including study center (grouped by country), age at blood draw, fasting status, date and time of blood draw, menopausal status at blood, menstrual cycle phase for premenopausal women at blood, OC/HRT use at blood, length of follow up. HRT use and hysterectomy are each additionally adjusted for the other. P values based on continuous variables; ‡p difference between pre- and postmenopausal: CA125 = 0.01; CA15.3 = 0.10; HE4 = 0.72; § Trend among parous women; ¶time between menarche and menopause with time subtracted for oral contraceptive use, pregnancy and breastfeeding; categories based on quartile cutpoints | | | | | | | | | | | |
